# Cohort event monitoring of booster COVID-19 vaccine safety using patient-reported outcomes in pregnant women

**DOI:** 10.3389/fdsfr.2025.1689349

**Published:** 2025-12-29

**Authors:** Emeline Maisonneuve, Nicoletta Luxi, Chiara Bellitto, Francesco Ciccimarra, Monika Raethke, Florence van Hunsel, Thomas Lieber, Erik Mulder, Fabio Riefolo, Felipe Villalobos, Nicolas H. Thurin, Francisco Batel-Marques, Kathryn Morton, Fergal O'Shaughnessy, Brian Cleary, Simona Sonderlichová, Andreea Farcas, Camelia Bucsa, Guillaume Favre, Satu J. Siiskonen, David Baud, Miriam C. J. M. Sturkenboom, Gianluca Trifirò, Alice Panchaud

**Affiliations:** 1 Materno-fetal and Obstetrics Research Unit, “Woman-Mother-Child” Department, Lausanne University Hospital, Lausanne, Switzerland; 2 Institute of Primary Healthcare (BIHAM), University of Bern, Bern, Switzerland; 3 Obstetrics Department, University Hospital, Zürich, Switzerland; 4 Department of Medicine, University of Verona, Verona, Italy; 5 Department of Diagnostics and Public Health, University of Verona, Verona, Italy; 6 Netherlands Pharmacovigilance Centre Lareb, ’s-Hertogenbosch, Netherlands; 7 Department of PharmacoTherapy, Epidemiology and Economics, Groningen Research Institute of Pharmacy (GRIP), University of Groningen, Groningen, Netherlands; 8 Teamit Institute, Partnerships, Barcelona Health Hub, Barcelona, Spain; 9 Fundació Institut Universitari per a la recerca a l’Atenció Primària de Salut Jordi Gol i Gurina (IDIAPJGol), Barcelona, Spain; 10 Bordeaux PharmacoEpi, INSERM CIC-P 1401, University Bordeaux, Bordeaux, France; 11 Laboratory of Social Pharmacy and Public Health, School of Pharmacy, University of Coimbra, Coimbra, Portugal; 12 Drug Safety Research Unit, Southampton, United Kingdom; 13 University of Portsmouth, Portsmouth, United Kingdom; 14 Pharmacy Department, Rotunda Hospital, Dublin, Ireland; 15 School of Pharmacy and Biomolecular Sciences, RCSI University of Medicine and Health Sciences, Dublin, Ireland; 16 Faculty of Medicine, SLOVACRIN, Pavol Jozef Šafárik University in Košice, Košice, Slovakia; 17 Pharmacovigilance Research Center, Iuliu Hatieganu University of Medicine and Pharmacy, Cluj-Napoca, Romania; 18 Division of Pharmacoepidemiology and Clinical Pharmacology, Utrecht Institute for Pharmaceutical Sciences (UIPS), Utrecht University, Utrecht, Netherlands; 19 Department of Data Science and Biostatistics, University Medical Centre Utrecht, Utrecht, Netherlands; 20 Pharmacy Service, Lausanne University Hospital, Lausanne, Switzerland

**Keywords:** adverse drug reaction, Covid booster, COVID vaccine, COVID-19, pregnancy, safety

## Abstract

**Background:**

COVID-19 vaccination is still accepted by only half of pregnant women. Further scientific evidence on the safety of the COVID-19 vaccine could help overcome vaccine hesitancy in this population.

**Objectives:**

The aim of this study is to describe and compare the incidence of solicited and unsolicited adverse drug reactions (ADRs) and serious adverse reactions following the booster dose of different COVID-19 vaccines in pregnant women versus the general female population.

**Methods:**

We conducted a web-based prospective observational cohort study. In eight European countries, pregnant women and non-pregnant women from the same source population who received a first or second booster dose of COVID-19 vaccine between February 2021 and February 2023 were included in the study if they registered within 48 h after vaccination and completed at least the baseline questionnaire and one follow-up questionnaire. The exposure was a mRNA COVID-19 vaccine of different brands used for a booster dose. The proportion of solicited and unsolicited ADRs following the booster dose by vaccine brand was computed across participating countries in pregnant and non-pregnant women. We performed descriptive analyses of medical characteristics of vaccinees. We compared the proportions of local and systemic solicited ADRs and unsolicited ADRs in pregnant vaccinees with a random sample (1:4 ratio) of age- and vaccine brand-matched vaccinees from the general female population.

**Results:**

Overall, 358 pregnant women were matched with 1,432 women from the general population. The proportion of women who reported at least one solicited adverse event was significantly lower in the pregnant group in the non-pregnant group (56.4% [95%CI 51.3%–61.5%] versus 72.3% [95%CI 70.0%–74.6%], p = 0.01). Regarding local ADRs, pregnant women reported significantly less pain and swelling. As for systemic ADRs, pregnant women reported all symptoms less frequently than non-pregnant women, except nausea. The proportion of women who reported at least one unsolicited adverse event was less frequent in the pregnant group (7.8% [95%CI 5.0%–10.6%] versus 24.9% [95%CI 22.7%–27.1%], p = 3.10^−10^). Serious adverse events were reported in 0.8% and 0.3% of pregnant and non-pregnant women respectively.

**Conclusion:**

This prospective study on COVID-19 vaccine safety monitoring showed that pregnant women from the general population reported solicited and unsolicited ADRs less frequently than non-pregnant women.

## 1 Introduction

Pregnant women with SARS-CoV-2 infection are at higher risk of maternal morbidity and mortality compared to the general female population, and their children are at higher risk of hospitalization in neonatal intensive care (Smith et al., 2023). There is growing evidence that the COVID-19 vaccine does not increase adverse obstetric and neonatal outcomes (Fernandez-Garcia et al., 2024; Shimabukuro et al., 2021a; Ciapponi et al., 2023; de Feijter et al., 2024; Woestenberg et al., 2023; Woestenberg et al., 2025). Moreover, COVID-19 vaccination during pregnancy is associated with a decrease of SARS-CoV-2 infection, stillbirth, and hospitalization in neonatal intensive care (Prasad et al., 2022; Halasa et al., 2022). Despite evidence of effectiveness and safety of COVID-19 vaccines, vaccination hesitancy remains widespread worldwide (Shamshirsaz et al., 2022; Nindrea et al., 2022; Azami et al., 2022; Bianchi et al., 2022; Razzaghi et al., 2023). The strongest determinant of vaccination willingness in pregnant women is the belief that the COVID vaccine is safe (Maisonneuve et al., 2023). Therefore, it is still necessary to provide more accurate scientific evidence on COVID-19 vaccine safety, including for booster doses, to overcome vaccination hesitancy among this special obstetric population. As most pregnant women are already immunized against COVID-19 through the first cycle of vaccination or have immunity due to previous infection, it is recommended to vaccinate them with an updated booster dose of mRNA vaccine during pregnancy (Garabedian et al., 2024; ACOG, 2024; Panagiotakopoulos et al., 2024).

In 2021, the European Medicines Agency (EMA) launched and funded a comprehensive multi-country project titled “COVID Vaccine Monitor” (CVM). The CVM is a large-scale cohort event monitoring study that aimed to collect self-reported outcomes from vaccinees regarding the safety of all EMA-authorized COVID-19 vaccines from the general population and special cohorts (children and adolescents, immunocompromised persons, persons with allergy, persons with a history of COVID-19, and pregnant and lactating women) (Ahmadizar et al., 2023; Bellitto et al., 2024; Ciccimarra et al., 2024). This active cohort event monitoring system provided the ability to monitor vaccine safety in the general population as well as special population cohorts in real time and to generate risk estimates.

The primary aim of this study was to describe and compare the incidence of solicited and unsolicited patient-reported adverse drug reactions (ADRs), adverse events of special interest (AESIs), and serious ADRs following the first or second booster dose of different mRNA COVID-19 vaccines in pregnant women versus the general female population across the participating countries between August 2021 and February 2023. The secondary aims were to compare the ADRs between brands and to describe maternal, obstetric, and neonatal outcomes of women who received a booster mRNA vaccine during pregnancy, overall and stratified by vaccine brand.

## 2 Materials and methods

### 2.1 Study design and setting

This was a prospective observational international cohort study. On the national level, data were prospectively collected in near real-time, directly from a cohort of vaccine recipients in different countries. Two data collection tools were used: Lareb Intensive Monitoring (LIM) web app, managed by the Netherlands Pharmacovigilance Centre Lareb, and Research Online (RO) web app, managed by the Julius Centre at University Medical Center in Utrecht. Once participants had been invited to participate, via e-mail or flyers, they registered themselves and created a study account on a website that was specifically designed for this study for each country in the local language(s). Participants used the LIM web app and/or the RO web app to access their questionnaires online and received reminders to fill them in. People registered only if they had received their booster dose within 48 h. Vaccinees were asked to fill in a questionnaire at baseline for their baseline characteristics as well as five follow-up questionnaires (at 1 week (Q1), 3 weeks (Q2), 5 weeks (Q3), 8 weeks (Q4), and 3 months (Q5) after the booster dose) collecting information on suspected ADRs possibly related to COVID-19 vaccines. Moreover, pregnant women received an additional specific “End of Pregnancy” questionnaire within 45 days after the estimated delivery due date to collect information on outcomes related to their pregnancy and their infant(s). Pregnant women could only register through the RO web app, since specific pregnancy-related questionnaires were built in this web app for the CVM project.

### 2.2 Study population

#### 2.2.1 Inclusion criteria

Pregnant and non-pregnant women who received a first or second booster dose of COVID-19 vaccine, registered within 48 h from the vaccine administration, provided informed consent, and completed the baseline questionnaire and at least one follow-up questionnaire were included in the analysis.

The cohort of pregnant women included persons vaccinated with a COVID-19 booster at any point of their pregnancy. Countries including pregnant women vaccinated with the booster between August 2021 and February 2023. Were France, Ireland, Italy, Portugal, Romania, Spain, Switzerland, and the UK

The matched non-pregnant women were women from the same source populations. As a control group, we selected a random sample of female vaccinees participating in the CVM project who registered within 48 h from the vaccine administration and did not report being pregnant. These women were matched to pregnant vaccinees based on vaccine brand and age but not based on country. They were vaccinated with the booster from February 2021 to February 2023.

#### 2.2.2 Exclusion criteria

Male vaccinees were excluded. Women under 18 years old were excluded from the group of pregnant women in Ireland and Switzerland. Otherwise, vaccinees could participate at any age (based on vaccination strategy) and minors (as defined by the laws of each country) who could participate via a proxy (a parent or legal representative) who would register and complete the questionnaires on their behalf were included in the children and adolescents’ cohorts.

### 2.3 Study variables

#### 2.3.1 Exposure

The vaccine brand of the first or second booster dose was collected: mRNA vaccines (Comirnaty - BioNTech Pfizer, Spikevax - Moderna) or an unknown brand.

#### 2.3.2 Covariates

For all vaccinees, age, height and weight, country, and medical history including information on comorbidities and concomitant diseases (e.g., diseases or drugs affecting the immune system, history of allergy, or prior SARS-CoV-2 prior infection) were collected. For pregnant women, baseline variables were collected regarding pregnancy (e.g., gravidity, parity, previous pregnancy complications, ongoing pregnancy due date, gestational age at vaccination, etc.).

#### 2.3.3 Outcome variables

##### 2.3.3.1 Solicited adverse drug reactions

Solicited ADRs were collected via closed dedicated questions. Local reactions at injection site included redness, warmth, pain, itching, hematoma, swelling, induration, and extensive limb swelling. Systemic reactions included fever/feverishness, shivering/chills, headache, nausea, myalgia/muscle pain, arthralgia/joint pain, malaise, and fatigue. The same solicited ADRs were collected across all participating countries, and they could be automatically MedDRA-coded.

##### 2.3.3.2 Unsolicited adverse drug reactions

In addition to solicited ADRs, the vaccinees were asked to report as a free text whether any other suspected ADR occurred (unsolicited). Pharmacovigilance assessors in the different participating countries coded unsolicited reported ADRs into MedDRA lower-level terms (in English) (MedDRA, 2025). The patients were also asked if they had COVID-19 during the 3 months after booster vaccination. The later follow-up periods served to monitor suspected adverse reactions with a longer lag time and to assess the course of previously reported adverse reactions (i.e., outcome and duration of symptoms).

##### 2.3.3.3 Adverse events of special interest

Adverse events are defined as undesirable experiences associated with the use of a medical product such as a vaccine. The Council for International Organizations of Medical Sciences (CIOMS) defines an AESI as a scientific and medical concern specific to the sponsor’s product or program, for which ongoing monitoring and rapid communication by the investigator to the sponsor could be appropriate (Younus et al., 2020). Such an event might require further investigation in order to characterize and understand it. AESIs for COVID-19 vaccines were defined according to a list provided by the Brighton Collaboration (Brighton, 2025).

##### 2.3.3.4 Serious adverse drug reactions

Pharmacovigilance assessors in the different participating countries determined whether ADRs were serious based on the criteria of the CIOMS criteria (Younus et al., 2020). The qualified assessors considered all information including possible uploads of documents by participants or comments on these events. If consent had been given by a participant, follow-up was requested by e-mail for verification and grading of the clinical documentation. Otherwise, serious ADR assessment was carried out by the Regional Center of Pharmacovigilance or local Pharmacovigilance Responsible Person, in agreement with national pharmacovigilance practice and legislation. There were thus two variables regarding the seriousness of an ADR: the seriousness which was reported by the participant and the seriousness as assessed by the trained assessors. Seriousness used in all tables was based on the assessed seriousness.

##### 2.3.3.5 Pregnancy and neonatal outcomes

After the end of pregnancy (45 days after the estimated due date, which was considered as 40 weeks after the last menstrual period), a dedicated questionnaire was sent to the women to collect key information on pregnancy and neonatal outcomes: pregnancy outcomes (miscarriage defined as the loss of the fetus before 20 weeks of pregnancy, livebirth, termination of pregnancy, or stillbirth defined as loss of the fetus after 20 weeks of pregnancy); pregnancy complications (gestational diabetes, hypertension, thrombosis, preeclampsia, intrauterine growth restriction, abnormal fetal Doppler, threatened preterm labor, placenta praevia, preterm premature rupture of membranes, or placental abruption); gestational age at the end of pregnancy (in weeks since last menstrual period); preterm birth defined as a delivery before 37 weeks of gestation; and neonatal outcomes (birthweight, physical examination abnormality, neonatal intensive care unit (NICU) admission, neonatal complications, neonatal death defined as death of the neonate in the first 30 days, and if the infant was still healthy at 30 days of life).

### 2.4 Statistical analysis

The common core data from different countries was pooled, stratified by special cohorts, and analyzed at the European level, using a common data model (CDM) (Luxi et al., 2023; Raethke et al., 2024).

#### 2.4.1 Frequency of ADRs

The incidences of patient-reported local and systemic solicited ADRs, as well as unsolicited and serious ADRs, following the first or second booster doses of different COVID-19 vaccines, stratified by age and vaccine brand, were measured as the proportion of the number of ADRs over the total number of subjects in the cohorts.

Categorical variables were reported as absolute frequencies and percentages, while continuous variables were reported as means (along with standard deviations, minimum and maximum). For continuous variables, the Shapiro-Wilk test was performed to assess the normal distribution. In the case of non-normality, medians and interquartile ranges were reported instead of means and standard deviations. A Chi-square test or Fisher exact test was performed to compare categorical variables as appropriate and Student t test or Mann Whitney U test was used to compare continuous variables as appropriate. A p-value<0.05 denotes statistical significance.

#### 2.4.2 Propensity score matching analysis

To compare the incidence of ADRs in vaccinated pregnant women versus non-pregnant women in the general population, the propensity score (PS) methodology was applied (Sturmer et al., 2014). PS was calculated from a logistic regression model predicting the individual probability of being in the group of pregnant women according to the following subject characteristics (covariates): age at study registration (± 1 year) and vaccine brand. Women vaccinated during pregnancy were then 1:4 matched to non-pregnant women on the basis of their PS value, using the nearest neighbor matching procedure (Gu and Rosenbaum, 1993). This 1:4 ratio was chosen as the best trade-off between increasing the statistical power and maintaining comparability in terms of age and vaccine brand distribution.

#### 2.4.3 Missing values

All participants with available baseline and at least Q1 were included in the analyses. Within this study population, some variables contained missing values because not all questionnaire items were mandatory. Therefore, the proportions for pregnancy and neonatal outcomes were calculated based on the available data for each specific variable, without excluding any participant from the analyses. The absolute counts of participants with missing values were displayed for each outcome.

## 3 Results

Overall, between February 2021 and February 2023, 358 pregnant women were recruited and matched with 1,432 non-pregnant women from the same source population, both populations having received a COVID-19 booster vaccine. Matching by PS allowed a balanced distribution of the matching factors. The two cohorts were vaccinated in the same proportions: 68.2% with Pfizer, 31.6% with Moderna, and 0.2% with a booster vaccine of unknown brand. The median age was 35 (interquartile range [IQR] 32–37) for pregnant women and 36 (IQR 31–41) for non-pregnant women. However, the breakdown by age category was different among both cohorts: 81.6% of pregnant women were aged ‘30–39’ followed by 10.6% aged ‘40–49’, while 50.6% of non-pregnant women were aged ‘30–39’ and 31.3% aged ‘40–49’. Pregnant women were recruited mainly in Ireland, Switzerland, and France, and matched non-pregnant women were recruited mainly in France, Italy, and the UK (Supplementary Figure S1). Medical history by organ system was comparable between pregnant and non-pregnant women, except for mental health disorders, which were lower in pregnant women (1.7% versus 4.7%, p = 0.01). The proportions of women with at least one medical condition were comparable in both cohorts (42.5% and 37.2%, p = 0.15) (Table 1; Supplementary Table S1). More pregnant women (1.7%) than non-pregnant women (0.3%) participated in the study after the second booster. The first questionnaire (Q1) was completed 1 week after the booster injection by 358 pregnant and 1,432 non-pregnant women. The last questionnaire (Q5) was completed 3 months after booster injection by 208 pregnant and 572 non-pregnant women (58.1% and 39.9% of those who answered Q1, respectively). The “end of pregnancy” questionnaire, sent 45 days after the estimated date of delivery, was completed by 55.9% (n = 200) of pregnant women (Supplementary Figure S2).

**TABLE 1:** Baseline characteristics of pregnant women and matched female adults who received a COVID-19 booster injection.

​	Pregnant cohort N = 358		Matched non-pregnant cohort N = 1,432		p-value
	n	%	n	%	
Vaccine brand					
BioNTech pfizer	244	68.2	976	68.2	​
Moderna	113	31.6	452	31.6	Matching
Unknown	1	0.2	4	0.2	Factor
Number of vaccine doses before the booster					
One cycle (two doses)	352	98.3	1,427	99.7	0.004
Three doses	6	1.7	5	0.3	​

Age	Median	IQR	Median	IQR	p-value
In years	35	32–37	36	31–41	Matching factor

​	n	%	n	%	p-value
Age category					
18–24 years	2	0.6	8	0.6	​
25–29 years	26	7.3	250	17.5	​
30–39 years	292	81.6	724	50.6	Matching
40–49 years	38	10.6	448	31.3	Factor
50–59 years	0	0.0	1	0.1	*p = 3.10* ^*−* *26*^
70–79 years	0	0.0	1	0.1	​
Country of residence					
France	37	10.3	726	50.7	​
Ireland	163	45.5	10	0.7	​
Italy	59	16.5	414	28.9	​
Portugal	10	2.8	21	1.5	<10^−16^
Romania	3	0.8	48	3.4	​
Spain	3	0.8	54	3.8	​
Switzerland	81	22.6	16	1.1	​
United Kingdom	2	0.6	143	10.0	​
Medical history (MedDRA PT)					
At least one medical condition	152	42.5	532	37.2	0.15
Cardiovascular diseases	2	0.6	19	1.3	0.23
Diabetes mellitus	6	1.7	11	0.8	0.11
Immunosuppression	4	1.1	37	2.6	0.10
Hypertension	4	1.1	24	1.7	0.45
Liver diseases	1	0.3	21	0.3	0.84
Lung diseases	21	5.9	77	5.4	0.72
Mental disorders	6	1.7	67	4.7	0.01
Malignant tumors	0	0	4	0.3	0.32
Neurological diseases	2	0.6	8	0.6	1
Renal diseases	1	0.3	7	0.5	0.60

Abbreviations: MedDRA, medical dictionary for regulatory activities; PT, preferred term. At least one medical condition means at least one of the medical conditions by organ system described in the table.

### 3.1 Solicited adverse drug reactions

The proportion of women who reported at least one solicited adverse event was significantly lower in the pregnant group: 56.4% (95%CI 51.3%–61.5%) compared to 72.3% (95%CI 70.0%–74.6%) in the non-pregnant group (p = 0.01). Regarding local solicited ADR, pregnant women reported significantly less pain (38.8%, [95%CI 33.8–43.8] versus 48.0%, [95%CI 45.4%–50.6%], p = 0.02) and less swelling (10.1%, [95%CI 7.0%–13.2%] versus 19.6%, [95%CI 17.5%–21.7%], p = 0.0001) than non-pregnant women. As for systemic solicited ADRs, pregnant women reported all symptoms (i.e., arthralgia, chills, fatigue, headache, malaise, myalgia, increased body temperature, and pyrexia) significantly less frequently than non-pregnant women, except nausea, for which there were no differences between the two groups (Table 2; Supplementary Figure S3). Moreover, solicited ADRs were significantly more frequent after Moderna than Pfizer/BioNtech vaccines for pregnant (69% versus 50.8%, p = 0.001) and non-pregnant women (77.4% versus 69.8%, p = 0.003) (Supplementary Table S2).

**TABLE 2:** Comparison of solicited local and systemic adverse drugs reactions between pregnant and matched non-pregnant women after booster injection.

​	Pregnant cohort N = 358		95% CI	Matched non-pregnant cohort N = 1,432		95% CI	p-value
	n	%		n	%		
At least one solicited ADR							
Yes	202	56.4	51.3–61.5	1,035	72.3	70.0–74.6	0.01
Local solicited ADR (MedDRA PT)							
Erythema	16	4.5	2.3–6.6	96	6.7	5.4–8.0	0.13
Hematoma	9	2.5	0.9–4.1	45	3.1	2.2–4.1	0.54
Induration	1	0.3	0–0.8	10	0.7	0.3–1.1	0.37
Inflammation	48	13.4	9.9–16.9	248	17.3	15.4–19.3	0.10
Pain	139	38.8	33.8–43.8	687	48.0	45.4–50.6	0.02
Pruritus	10	2.8	1.1–4.5	66	4.6	3.5–5.7	0.14
Local reaction^a^	0	0	0–0	2	0.1	0–0.3	0.48
Swelling	36	10.1	7.0–13.2	280	19.6	17.5–21.7	0.0001
Warmth	27	7.5	4.8–10.3	101	7.1	5.7–8.4	0.76
Systemic solicited ADR (MedDRA PT)							
Arthralgia	39	10.9	7.7–14.1	223	15.6	13.7–17.5	0.04
Chills	39	10.9	7.7–14.1	334	23.3	21.1–25.5	4.10^–6^
Fatigue	105	29.3	24.6–34.1	607	42.4	39.8–45.0	0.0005
Headache	84	23.5	19.1–27.9	497	34.7	32.2–37.2	0.0008
Malaise	73	20.4	16.2–24.6	418	29.2	26.8–31.5	0.004
Myalgia	65	18.2	14.2–22.2	462	32.3	29.8–34.7	10^–5^
Nausea	38	10.6	7.4–13.8	168	11.7	10.1–13.4	0.58
Body temperature increased^b^	14	3.9	1.9–5.2	104	7.3	5.9–8.6	0.03
Pyrexia^c^	18	5.0	2.8–7.3	181	12.6	10.9–14.4	0.0001
Hyperpyrexia^d^	0	0	0–0	0	0	0–0	#

Abbreviations: ADR, adverse drug reaction; ICU: intensive care unit; MedDRA = medical dictionary for regulatory activities; PT = preferred term.

^a^ Local injection site reaction is defined as two or more of the following adverse reactions (redness, warmth, pain, swelling).

^b^ Body temperature increased is defined as body temperature between 37.5 and 37.9 °C.

^c^ Pyrexia is defined as body temperature between 38.0 and 40.4 °C.

^d^ Hyperpyrexia as body temperature at 40.5 and 42.0 °C.

^#^Chi^2^ test could not be performed because when cells did not contain positive numbers.

### 3.2 Unsolicited adverse drug reactions

The pregnant women reported at least one unsolicited adverse event less frequently (7.8%, [95%CI 5.0–10.6]) than the non-pregnant women (24.9%, [95%CI 22.7–27.1]) (p = 3.10^−10^). The list of unsolicited ADRs among pregnant women and matched non-pregnant women after booster injection i) after all vaccines combined and ii) stratified by vaccine brand is displayed in Supplementary Table S3. Unsolicited ADRs were similar after Pfizer/BioNtech and Moderna vaccines for pregnant (7.4% versus 8.8%, p = 0.63) and non-pregnant women (24.9% versus 25.0%, p = 0.91) (Supplementary Table S3).

Mental health disorders were significantly associated with solicited ADRs (p = 0.005) and tended to be associated with unsolicited ADRs (p = 0.06) (Supplementary Table S4).

### 3.3 Adverse reactions of special interest

Pregnant women did not report any AESI. However, six AESI were reported in the matched non-pregnant women: arrhythmia (n = 2), COVID-19 (n = 2), and hypersomnia (n = 1) after Pfizer/BioNtech booster vaccine and arrhythmia (n = 1) after Moderna (Table 3; Supplementary Table S3).

**TABLE 3:** Comparison of unsolicited adverse drugs reactions, adverse events of special interest, and serious adverse outcomes between pregnant and matched non-pregnant women after booster injection.

​	Pregnant cohort N = 358		Matched non-pregnant cohort N = 1,432		p
	n	%	n	%	
Unsolicited ADR					
At least one unsolicited ADR^a^	28/358	7.8	356/1,432	24.9	3.10^–10^
Questionnaire mentioning unsolicited ADR	​	​	​	​	​
Q1 (1 week)	21/358	5.9	277/1,432	19.3	​
Q2 (3 weeks)	6/316	1.9	59/1,152	5.1	​
Q3 (5 weeks)	3/283	1.1	42/939	4.5	​
Q4 (8 weeks)	2/252	0.8	14/657	2.1	​
Q5 (3 months)	0/208	0	7/572	1.2	​
Dose of booster	​	​	​	​	​
Unsolicited ADR after first booster	27/352	7.6	353/1,427	24.7	5.10^–10^
Unsolicited ADR after second booster	1/6	16.7	3/5	60.0	0.25
Adverse event of special interest					
At least one adverse event of special interest^b^	0/358	0	6/1,432	0.4	^e^
Questionnaire mentioning AESI	​	​	​	​	​
Q1 (1 week)	0/358	0	3/1,432	0.2	​
Q2 (3 weeks)	0/316	0	2/1,152	0.2	​
Q3 (5 weeks)	0/283	0	0/939	0	​
Q4 (8 weeks)	0/252	0	1/657	1.5	​
Q5 (3 months)	0/208	0	0/572	0	​
Serious ADRs					
At least one serious ADR^c^	3/358	0.8	5/1,532	0.3	0.08
Questionnaire mentioning serious ADR	​	​	​	​	​
Q1 (1 week)	1/358	0.3	4/1,432	0.3	​
Q2 (3 weeks)	0/316	0	1/1,152	0.1	​
Q3 (5 weeks)	1/283	0.4	0/939	0	​
Q4 (8 weeks)	1/252	0.4	1/657	0.2	​
Q5 (3 months)	0/208	0	0/572	0	​
At least one serious maternal ADR^d^	1/358	0.3	5/1,532	0.3	1
Maternal severe adverse outcomes					
Hospitalization possibly related to vaccination	0	0	0	0	^e^
Maternal ICU admission	0	0	0	0	^e^
Maternal death	0	0	0	0	^e^

^a^ The reported unsolicited ADRs, are displayed in Supplementary Table S3.

^b^ The reported unsolicited adverse reactions of special interest (AESI) were arrhythmia (n = 3), COVID-19 (n = 2), and hypersomnia for matched non-pregnant women.

^c^ The reported serious adverse reactions were congenital anomaly, hemorrhage, and loss of consciousness for pregnant women and gastritis, headache, nausea, pyrexia (n = 2), and tachycardia for matched non-pregnant women.

^d^ Only maternal serious ADRs, are compared, excluding fetal malformation or pregnancy-specific events, as these cannot occur in the control group.

^e^ Chi^2^ test could not be performed because cells did not contain positive numbers.

### 3.4 Serious adverse ADRs

Among the 358 pregnant women, three (0.8%) serious ADRs were reported. The first serious ADR was a congenital anomaly reported on Q4, which was a fetal bladder exstrophy; the gestational age at vaccination was not specified by the vaccinee. The second serious ADR was a hemorrhage in pregnancy reported on Q3, but the subject did not fill the end of pregnancy questionnaire. Therefore, no information is available on the pregnancy outcome. The third serious ADR was a loss of consciousness reported on Q1. These three ADRs occurred after the Pfizer/BioNtech booster vaccine. Among the 1,432 non-pregnant women, five (0.3%) serious ADRs were reported (tachycardia reported on Q1, headache reported on Q1, nausea reported on Q1, pyrexia reported on Q1 and Q2 (n = 1), and gastritis reported on Q4 after the Pfizer/BioNtech booster vaccine and pyrexia reported on Q1 after Moderna (n = 1)). The difference in rates of serious ADRs between pregnant and non-pregnant women was not statistically significant (p = 0.08).

### 3.5 Maternal, obstetric, and neonatal outcomes

Baseline sociodemographic and obstetric characteristics are described in Table 4. The median of the gestational age at vaccination was 24 weeks (IQR: 12–28 weeks). Among 200 women (55.9% of the cohort) who answered the ‘end of pregnancy’ questionnaire, there were 98.5% livebirths, no stillbirths, no ectopic pregnancies, and 1.5% miscarriages (Table 5). The miscarriages occurred at six, seven, and 9 weeks of gestation, respectively. Among livebirths, the median gestational age at delivery was 39 weeks (IQR 38–40) and was similar for pregnant women vaccinated with Pfizer/BioNTech and Moderna. Preterm birth occurred in 3.0% of livebirths (one case at 33 weeks, one case at 34 weeks, and four cases at 36 weeks). The rates of gestational diabetes, preeclampsia, and intrauterine growth were 10.2%, 3.0%, and 4.1%, respectively. Maternal, obstetric, and neonatal outcomes were comparable between the two brands of vaccine.

**TABLE 4:** Baseline sociodemographic and self-reported obstetric characteristics of pregnant women according to COVID vaccine brand.

Gestational age at vaccination	All vaccines N = 358^b^		Pfizer/BioNTech N = 244		Moderna N = 113	
	Median	IQR	Median	IQR	Median	IQR
Gestational age (in wks)^a^	24.0	12.0–28.0	18.5	7.0–29.25	25.0	18.0–27.0
	Min-max	5.0–38.0	Min-max	5.0–38.0	Min-max	12.0–35.0

​		n	%	n	%	n	%
Trimester at vaccination							
1st trimester (<14 weeks)		5	38.4	4	50.0	1	20.0
2nd trimester (14^0/7^ wks −27^6/7^ wks)		4	30.8	1	12.5	3	60.0
3rd trimester (28^0/7^ wks - delivery)		4	30.8	3	37.5	1	20.0
*Missing data*		*345*	—	*236*	-	*108*	—
Obstetric history							
Gravidity	G1	174	48.6	113	46.3	61	54.0
	G2	100	27.9	73	29.9	26	23.0
	G3	48	13.4	33	13.5	15	13.3
	≥ G4	36	10.1	25	10.2	11	9.7
	*Missing data*	*0*	—	*0*	—	*0*	—
Number of previous pregnancies ≥39 weeks^c^	0	35	19.6	26	19.8	9	17.3
	1	114	63.7	83	63.4	31	59.6
	2	29	16.2	20	15.3	9	17.3
	≥3	1	0.6	0	0	1	1.9
	*Missing*	*5*	—	*2*	—	*3*	—
Number of previous preterm^d^ pregnancies^c^	*0*	135	86.5	95	72.5	39	75.0
	*1*	20	12.8	15	11.5	5	9.6
	*2*	1	0.6	1	0.8	0	0
	*Missing*	*28*	—	*20*	—	*8*	—
History of stillbirth^c^	*Yes*	3	4.3	3	6.3	0	0
	*No*	66	95.7	45	93.8	21	100
	*Missing*	*115*	—	*83*	—	*31*	—
Obstetric baseline characteristics							
Number of fetuses	Singleton	290	97.6	201	97.6	88	97.8
	Multiple	7	2.4	5	2.4	2	2.2
	*Not sure*	*13*	—	*10*	—	*3*	—
	*Missing*	*48*	—	*28*	—	*20*	—
Diabetes mellitus	Preexisting	2	0.6	2	0.9	0	0
	Gestational	16	5.2	12	5.6	4	4.3
	No	292	94.2	202	93.5	89	95.7
	*Missing*	*48*	—	*28*	—	*20*	—
Hypertension	Chronic	4	1.3	4	1.9	0	0
	Gestational	7	2.3	4	1.9	2	2.2
	No	299	96.5	208	96.3	91	97.8
	*Missing*	*48*	—	*28*	​	*20*	—
Thrombosis	Pre-pregnancy	3	1.0	0	0	3	3.2
	During pregnancy	3	1.0	2	0.9	1	1.1
	No	304	98.0	214	99.1	89	95.7
	*Missing*	*48*	—	*28*	—	*20*	—

*«*G» stands for gravidity. Wks: weeks. The superscripts^0/7^ to^6/7^are the numbers of days in addition to the weeks of gestation.

^a^ Number of weeks at injection was calculated according to this formula: 40 - (Due date–Date of injection)/7

^b^ The total of vaccinated women was 358 (244 vaccinated with Pfizer BioNTech, 133 with Moderna, 1 vaccine brand unknown). The data related to the unknown vaccine brand are not displayed on this table.

Proportions are expressed considering only available data.

^c^ Counts and proportions are displayed only among pregnant women with at least one previous pregnancy.

^d^ Preterm birth is defined as a delivery before 37 weeks of gestation.

**TABLE 5:** Self-reported obstetric outcomes according to COVID vaccine brand among all participants with available obstetric outcomes.

​	All vaccine brands N = 200		Pfizer BioNTech N = 145		Moderna N = 55	
	n	%	n	%	n	%
Pregnancy outcome						
Live birth	197	98.5	143	98.6	54	98.2
Stillbirth^a^	0	0.0	0	0.0	0	0.0
Miscarriage^b^	3	1.5	2	1.4	1	1.8
Ectopic pregnancy	0	0.0	0	0.0	0	0.0

​	All vaccine brands N = 197		Pfizer BioNTech N = 143		Moderna N = 54	
	n	%	n	%	n	%
Maternal adverse outcome						
Thromboembolic event	0	0.0	0	0.0	0	0.0
Obstetric adverse outcome						
Gestational diabetes	20	10.2	12	8.4	8	14.8
Gestational hypertension	16	8.1	11	7.7	5	9.3
Preeclampsia	6	3.0	4	2.8	2	3.7
Threatened preterm labor	8	4.1	6	4.2	2	3.7
Placenta previa	2	1.0	2	1.4	0	0.0
Preterm rupture of membranes	3	1.5	3	2.1	0	0.0
Placental abruption	0	0.0	0	0.0	0	0.0
Preterm birth^c^	6	3.0	4	2.8	2	3.7

Gestational age at delivery	Median	IQR	Median	IQR	Median	IQR
Gestational age (in weeks)	39	38–40	39	38–40	39	38–40
*Unknown*	*4*	​	*3*	​	*1*	​

Fetal adverse outcome	n	%	n	%	n	%
Intrauterine growth restriction	8	4.1	5	3.5	3	5.5
Abnormal fetal Doppler	3	1.5	2	1.4	1	1.8

Birthweight	Mean	SD	Mean	SD	Mean	SD
Birthweight (grams)	3,499	515	3,560	532	3,350	444

​	All vaccine brands N = 192		Pfizer BioNTech N = 139		Moderna N = 53	
	n	%	n	%	n	%
Neonatal adverse outcomes						
Neonatal death^d^	0	0.0	0	0.0	0	0.0
NICU admission	0	0.0	0	0.0	0	0.0
Physical birth defect^e^	4	2.1	4	2.9	0	0.0
Neonatal infection	7	3.6	5	3.6	2	3.8
Neonatal hypoglycemia	9	4.7	6	4.3	3	5.7
Physical injury at birth	2	1.0	2	1.4	0	0.0
Feeding problems within 2 weeks	13	6.8	8	5.8	5	9.4
Hypothermia	2	1.0	1	0.7	1	1.9
Jaundice	54	28.1	43	30.9	11	20.8
Other conditions	9	4.7	7	5.0	2	3.8
None of these conditions	110	57.3	75	54.0	35	66.0
Healthy at 30 days	184	95.8	134	92.4	50	90.9
Not healthy at 30 days	8	4.2	5	3.4	3	5.5

The data related to the unknown vaccine brand is not displayed in this table.

Proportions are expressed considering only available data.

IQR: interquartile range; NICU: neonatal intensive care unit.

^a^ Stillbirth is defined as loss of the fetus after 20 weeks of pregnancy.

^b^ Miscarriage is defined as the loss of the fetus before 20 weeks of pregnancy.

^c^ Preterm birth is defined as a delivery before 37 weeks of gestation (after exclusion of the miscarriages and four instances of unavailable data).

^d^ Neonatal death is defined as death of the neonate in the first 30 days.

^e^ Abnormal findings were bladder exstrophy, and the three other cases were not described.

The ‘end of pregnancy questionnaire’ was not completed by 158 (44.1%) pregnant vaccinees. The determinants associated with lost-to follow-up were ‘age 25–29 years’ compared to ‘age 30–39 years’ and Italy as country of residence. The characteristics associated with completing the ‘end of pregnancy questionnaire’ are provided in Supplementary Table S5.

## 4 Discussion

To the best of our knowledge, this is the first cohort event monitoring that provided a comprehensive, comparative assessment of the safety of COVID-19 booster vaccines in pregnant women compared to a matched cohort of non-pregnant women from different European countries, within a prospective design. In this prospective observational study, the proportion of women who reported at least one solicited adverse event and at least one unsolicited adverse were less frequent in the pregnant group than the non-pregnant group (56.4% versus 72.3%, and 7.8% versus 24.9%, respectively). Interestingly, pregnant women reported all symptoms included in the systemic solicited ADRs significantly less frequently than non-pregnant women, except nausea.

Pregnancy is a unique immune condition that is modulated but not suppressed. In pregnant women, immunological adaptations occur, though the exact mechanisms remain unclear. The placenta likely plays a pivotal role as a potent immune-regulatory interface to create a tolerogenic environment (Mor and Cardenas, 2010; Abu-Raya et al., 2020). This may lead to less systemic ADRs in pregnant women than in the general female population. Our findings on solicited systemic ADRs, which are almost all decreased in pregnant women, are in line with the existing literature but not those on solicited local ADRs (decrease in pain and swelling in pregnant women from our study). First, regarding the first cycle of COVID-19 vaccination, using the V-safe pregnancy registry and the Vaccine Adverse Event Reporting System (VAERS), Shimabukuro et al. compared ADRs between pregnant and non-pregnant women between December 2020 and February 2021 in the United States (Maisonneuve et al., 2011). In their study, headache, myalgia, chills, and fever were reported less frequently among pregnant than non-pregnant women, whereas injection-site pain was reported more frequently (Shimabukuro et al., 2021b). Second, regarding the COVID-19 booster dose, Kachikis et al. found that pregnant participants were less likely to report any systemic reaction (adjusted odds ratio [aOR]: 0.7, [95%CI 0.6–0.8], p < 0.001) but more likely to report any local reaction to a COVID-19 booster or third dose compared with individuals who were neither pregnant nor lactating (aOR:1.2, [95%CI 1.0–1.4], p = 0.01). More specifically, in their study, pregnant participants in their third trimester at the time of the booster or third COVID-19 vaccine dose reported fewer systemic symptoms than pregnant participants in their first and second trimesters (Kachikis et al., 2022).

There are few studies on unsolicited ADRs after COVID-19 vaccination during pregnancy. Kachikis et al. did not describe specifically unsolicited ADRs, but they found that the most commonly reported obstetrics-related symptom within 24 h of receipt of a COVID-19 booster or third dose was contractions (11 of 2009 [0.6%]), which were reported only by participants in their third trimester (Kachikis et al., 2022). In our study, at least one unsolicited ADR was reported substantially less by pregnant women than by non-pregnant women (7.8% versus 24.9%). Mental disorders were the only significant baseline medical condition differing between the two groups. Mental disorders were associated with the reporting of solicited ADRs as well as a trend toward an association with unsolicited ADRs. However, the association between mental disorders and increased reporting of ADRs has not been documented in the literature so far.

The miscarriage rate in our study (1.5%) was lower than in the general pregnant population, likely because we did not follow pregnant vaccinees from the first trimester. Preterm birth occurred in 3.0% of live births, a rate notably lower than the 6.9% reported in Europe (EuroPeristat, 2025). This may be due to the higher socioeconomic status of pregnant women who choose vaccination and participate in online studies. Our rates of gestational diabetes, preeclampsia, and intrauterine growth restriction were 10.2%, 3.0%, and 4.1%, respectively. They are comparable with those from European studies in 2021 (11%, 2.3%, and 5.2%, respectively) (Leray et al., 2021; Paulo et al., 2021). Physical birth defects were reported in 2.1% of neonates in our study, which is comparable with 2.7% reported by EUROCAT in 2021-2022 (EUROCAT, 2025). There was no difference between maternal, obstetric, and neonatal outcomes between the two brands of vaccine, except for the mean birthweight, which was higher in neonates whose mother received the Pfizer/BioNTech vaccine than in those whose mothers had received the Moderna vaccine.

Since the start of the pandemic, the main driver of vaccination hesitancy is the belief that COVID-19 vaccines might not be safe (Maisonneuve et al., 2023). Our cohort event monitoring study showed that vaccination with the COVID-19 booster was safe, with less local and systemic ADRs in pregnant women than in non-pregnant women, no adverse event of special interest, and 0.8% of serious ADRs in pregnant women, which was comparable to those of the general population. These findings add evidence to the existing reassuring scientific literature to support vaccination against COVID-19 during pregnancy. Furthermore, the main reported vaccination-associated symptom that could induce obstetric complications and/or hospitalization is fever. In our study, pregnant women reported less increased body temperature and less pyrexia than non-pregnant women. There were no cases of hyperpyrexia in both groups. Moreover, fever could be mitigated with paracetamol in pregnant women.

One of the main strengths of this study lies in its inclusion of patient-level data from eight European countries, which were gathered and analyzed using a CDM. Furthermore, due to the flexibility of the LIM and RO web applications, it was possible to integrate and adapt them to the evolution of the pandemic and, considering the new information on vaccines made available during the study, facilitating the direct and timely updating of questionnaires. Another strength of the study is the inclusion within 48 h after vaccination and all follow-up questionnaires, which avoided recall bias. Lastly, this study was focused on COVID-19 mRNA booster dose only, which is currently recommended in many national guidelines for pregnant women. The boosters are being updated to adapt the new variants of Omicron, and the main mRNA vaccines recommended for pregnant women are still Pfizer/BioNTech and Moderna (ACOG, 2024; Swiss Federal Office of Public Health F, 2024).

However, we must acknowledge some limitations. The first limitation is the small number of vaccinees recruited and the 44% of participants who did not fill the end of pregnancy questionnaire, which prevents our study population from being representative of the population of vaccinated pregnant women. The design of the study with the inclusion within 48 h after the booster injection largely limited the recruitment of the vaccinees. Some vaccinees complained that the questionnaires were too long, and they sometimes had issues with the passwords, which discouraged them to log in to the website to complete the follow-up questionnaires. The high rate of lost-to-follow-up participants may have overestimated the prevalence of ADRs and adverse obstetrical outcomes. The second limitation concerns the comparative group, which was a cohort of female vaccinees who were not pregnant but could be part of other special populations in the study (e.g., with allergy, immunocompromised, having had a history of COVID-19 before the booster, or be lactating). They were also older despite matching based on age. This could have overestimated the prevalence estimates of reported ADRs in the comparison group. Moreover, pregnant and non-pregnant women mainly came from different countries: 68% of the pregnant group were living in Ireland or Switzerland, but less than 5% of the non-pregnant population were from these two countries and one-half were from France. These differences in country of residence may also influence the differential rates of reported outcomes between pregnant and non-pregnant women, due to different cultures, different considerations of pain, different relationships with the healthcare system and vaccination, and different media exposure regarding the COVID-19 vaccine and its potential AESI. Third, we performed multiple comparisons between pregnant and non-pregnant women, which may have led us to find statistical differences by chance. Fourth, the secondary outcomes on maternal, pregnancy, or neonatal complications were self-reported, and there was no control group without booster vaccination. Nevertheless, the obstetric complications were similar to those usually reported (EuroPeristat, 2025; Leray et al., 2021; Favre et al., 2022; European Medicines Agency, 2024). Finally, this study was unfortunately not able to provide accurate gestational age at the time of vaccination for all participants, which is crucial to analyze the potential impact of vaccination on obstetric adverse outcomes.

In 2024–25, American and European national guidelines still recommend vaccination against COVID-19 during pregnancy (Garabedian et al., 2024; ACOG, 2024; Swiss Federal Office of Public Health F, 2024). According to American College of Obstetrics and Gynecology (ACOG, 2024) guidelines, COVID-19 vaccines may be administered simultaneously with other vaccines. This includes vaccines routinely recommended during pregnancy, such as influenza, RSV, and Tdap (ACOG, 2024). Nevertheless, the US Centers for Disease and Control reported that, during the 2022–23 influenza season, 47.2% of women received influenza vaccination before or during pregnancy, 55.4% of women received Tdap vaccination during pregnancy, and 27.3% of women received a COVID-19 bivalent booster vaccine before or during pregnancy (Razzaghi et al., 2023). In the US (Wisconsin), 17.2% of persons who gave birth during the 2023–24 RSV season received the RSV vaccine during pregnancy (Kemp et al., 2025). A combination vaccine would help address hesitancy toward receiving multiple vaccinations, especially during the autumn-winter season (Licata et al., 2023). To overcome vaccine hesitancy, the ACOG guidelines recommend that obstetricians-gynecologists and other healthcare practitioners should lead by example by being vaccinated and encouraging eligible patients to be vaccinated as well (ACOG, 2024; Kachikis et al., 2022). Another benefit of COVID-19 vaccination is the protection against the risk of having long COVID, which happens in 10%–11% of cases after acute COVID-19 in the general population and during pregnancy (Davis et al., 2023; Yao et al., 2024). *In utero* exposure to mRNA-COVID-19 vaccine is not associated with an increased risk of impaired neurodevelopment at 12 months (Favre et al., 2025). Additional studies are needed to confirm these results, especially long-term evaluation of infant cognitive outcomes.

## 5 Conclusion

This prospective study on COVID-19 vaccine safety monitoring showed an overall favorable safety profile of COVID-19 vaccines in pregnant women within 3 months after injection, who reported solicited and unsolicited ADRs less frequently than non-pregnant women. Solicited ADRs were significantly more frequent after Moderna than Pfizer/BioNtech vaccines for pregnant women, and unsolicited ADRs were comparable for both brands. Pregnant women did not report any AESI. Serious adverse ADRs were reported in 0.8% and 0.3% in pregnant and non-pregnant women respectively. There was no signal for increased maternal, obstetrical, or neonatal poor outcomes. The findings of our study provide detailed data on the COVID-19 booster vaccine administered during pregnancy, which should help to overcome pregnant women’s hesitancy to be vaccinated.

## Data Availability

The raw data supporting the conclusions of this article will be made available by the authors, without undue reservation.
