# Supplementary material for: Cohort event monitoring of booster COVID-19 vaccine safety using patient-reported outcomes in pregnant women
Source: Front Drug Saf Regul. 2025 Dec 29;5:1689349. doi: 10.3389/fdsfr.2025.1689349 (PMC12791168; doi:10.3389/fdsfr.2025.1689349)
Supplement: Supplementary file 1 [file DataSheet1.docx]

**Cohort event monitoring of booster COVID-19 vaccine safety using patient-reported outcomes in pregnant women.**

Supplementary tables and figures

Table of Contents

[Supplementary figure 1: Distribution of the pregnant and matched non-pregnant women vaccinees among the eight European countries. 2](#_Toc215003451)

[Supplementary figure 2: Flowchart of questionnaires completed by pregnant and matched non-pregnant women who received a COVID-19 booster injection. 2](#_Toc215003452)

[Supplementary figure 3: Proportion of solicited adverse drugs reactions in pregnant and matched non-pregnant women who received a COVID-19 booster injection. 3](#_Toc215003453)

[Supplementary figure 4: Flowchart displaying the participating subjects by country. 3](#_Toc215003454)

[Supplementary table 1: Baseline characteristics of pregnant women and matched non-pregnant women after booster injection, overall and stratified by vaccine brand. 4](#_Toc215003455)

[Supplementary table 2: Comparison of solicited adverse drug reactions between pregnant women and matched non-pregnant women after booster injection, overall and stratified by vaccine brand. 5](#_Toc215003456)

[Supplementary table 3: List of unsolicited adverse drug reactions between pregnant women and matched non-pregnant women after booster injection, overall and stratified by vaccine brand. 7](#_Toc215003457)

[Supplementary table 4: Adverse drug reactions according to mental health disorders 12](#_Toc215003458)

[Supplementary table 5: Characteristics associated with completing the end of pregnancy questionnaire among pregnant women vaccinated with COVID-19 booster. 13](#_Toc215003459)

[Supplementary Table 6: Adverse drugs reactions (ADR) stratified by the number of comorbidities 14](#_Toc215003460)

[Supplementary Table 7: Adverse drugs reactions (ADR) stratified by country 15](#_Toc215003461)

# **Supplementary figure 1:** Distribution of the pregnant and matched non-pregnant women vaccinees among the eight European countries.

| 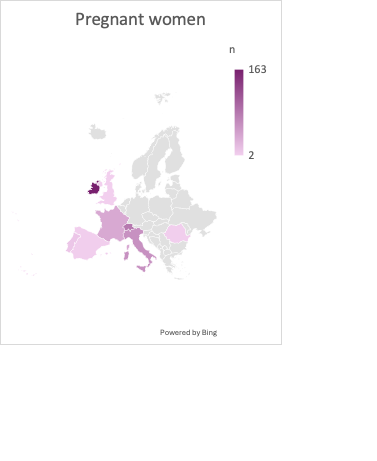 | **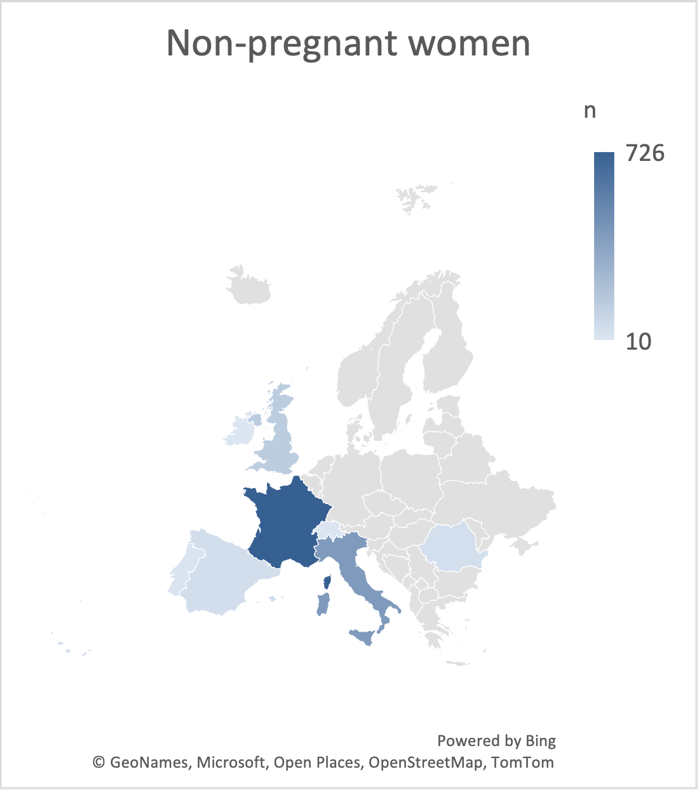** |
| --- | --- |

# **Supplementary figure 2:** Flowchart of questionnaires completed by pregnant and matched non-pregnant women who received a COVID-19 booster injection.

The questionnaires were completed at 1 week (Q1), 3 weeks (Q2), 5 weeks (Q3), 8 weeks (Q4), 3 months (Q5). End of pregnancy questionnaire was sent 45 days after the theorical due date of delivery (corresponding to 40 weeks of pregnancy).

# **Supplementary figure 3:** Proportion of solicited adverse drugs reactions in pregnant and matched non-pregnant women who received a COVID-19 booster injection.

ADR: adverse drug reaction; Injection site reaction is defined as 2 or more of the following adverse reactions (redness, warmth, pain, swelling) going past the elbow or shoulder.

# **Supplementary figure 4:** Flowchart displaying the participating subjects by country.

**
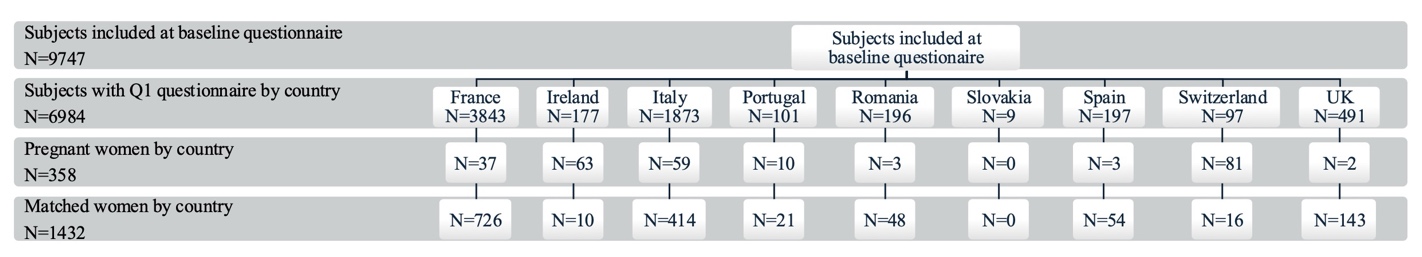
**

# **Supplementary table 1:** Baseline characteristics of pregnant women and matched non-pregnant women after booster injection, overall and stratified by vaccine brand.

| **COVID-19 vaccine brand** | **All vaccines** | | **Pfizer/BioNTech** | | **Moderna** | |
| --- | --- | --- | --- | --- | --- | --- |
|  | **Cohort**  **N=358** | **Matched**  **N=1,432** | **Cohort**  **N=244** | **Matched**  **N=976** | **Cohort**  **N=113** | **Matched**  **N=452** |
| **Median Age (IQR)** | 35 (32-37) | 36 (31–41) | 35 (32-37) | 36 (30-43) | 35 (32-37) | 35 (32-37) |
| **Body mass index category*, n (%)** | | | | | | |
| underweight | 7 (2.0) | 84 (5.9) | 6 (2.5) | 61 (6.3) | 1 (0.9) | 23 (5.1) |
| normal weight | 152 (42.5) | 849 (59.3) | 101 (41.4) | 571 (58.5) | 51 (45.1) | 278 (61.5) |
| overweight | 117 (32.7) | 279 (19.5) | 83 (34.0) | 196 (20.1) | 34 (30.1) | 82 (18.1) |
| obese | 68 (19.0) | 158 (11.0) | 42 (17.2) | 99 (10.1) | 25 (22.1) | 56 (12.4) |
| unknown | 14 (3.9) | 62 (4.3) | 12 (4.9) | 49 (5.0) | 2 (1.8) | 13 (2.9) |
| **Medical history (MedDRA PT), n (%)** | | | | | | |
| **At least one medical condition** | **152 (42.5)** | **532 (37.5)** | **102 (41.8)** | **387 (39.7)** | **50 (44.2)** | **143 (31.6)** |
| Cardiovascular disorder | 2 (0.6) | 19 (1.3) | 1 (0.4) | 14 (1.4) | 1 (0.9) | 5 (1.1) |
| Diabetes mellitus** | 6 (1.7) | 11 (0.8) | 4 (1.6) | 10 (1.0) | 2 (1.8) | 0 (0) |
| Hypertension | 4 (1.1) | 37 (2.6) | 2 (0.8) | 17 (1.7) | 2 (1.8) | 5 (1.1) |
| Immunosuppression | 4 (1.1) | 24 (1.7) | 2 (0.8) | 28 (2.9) | 2 (1.8) | 8 (1.8) |
| Liver disorder | 1 (0.3) | 21 (0.3) | 0 (0) | 3 (0.3) | 1 (0.9) | 2 (0.4) |
| Lung disorder | 21 (5.9) | 77 (5.4) | 12 (4.9) | 56 (5.7) | 9 (8) | 21 (4.6) |
| Mental disorder | 6 (1.7) | 67 (4.7) | 4 (1.6) | 46 (4.7) | 2 (1.8) | 21 (4.6) |
| Neoplasm malignant | 0 (0) | 4 (0.3) | 0 (0) | 4 (0.4) | 0 (0) | 0 (0) |
| Neurologic disorder | 2 (0.6) | 8 (0.6) | 2 (0.8) | 5 (0.5) | 0 (0) | 3 (0.7) |
| Renal disorder | 1 (0.3) | 7 (0.5) | 1 (0.4) | 6 (0.6) | 0 (0) | 1 (0.2) |

Abbreviations: ADR = adverse drug reaction; MedDRA=Medical Dictionary for Regulatory Activities; PT=preferred term

Baseline characteristics are displayed in absolute counts (percentages). If we consider all the vaccinees, there are 358 pregnant women matched with 1432 non-pregnant women. When we look at the brands, 244 pregnant women who received the Pfizer vaccine were matched with 976 non-pregnant women ; 113 women who received the Moderna vaccine were matched with 452 non-pregnant women ; and one woman who received a vaccine of unknown brand was matched with 4 non-pregnant women (these 5 women do not appear in the columns related to the brand).

*Body mass index was calculated based on the weight reported by the pregnant women at the time of vaccination and not based on their pre-pregnancy weight.

** The numbers of women with diabetes mellitus do not add up because one matched woman with diabetes was matched with the woman who received the vaccine if unknown brand.

#

# **Supplementary table 2:** Comparison of solicited adverse drug reactions between pregnant women and matched non-pregnant women after booster injection, overall and stratified by vaccine brand.

| **COVID-19 vaccine brand** | **All vaccines** |  |  |  | **Pfizer/**  **BioNTech** |  |  |  | **Moderna** |  |  |  |
| --- | --- | --- | --- | --- | --- | --- | --- | --- | --- | --- | --- | --- |
|  | **Cohort**  **N=358** |  | **Matched**  **N=1,432** |  | **Cohort**  **N=244** |  | **Matched**  **N=976** |  | **Cohort**  **N=113** |  | **Matched**  **N=452** |  |
| **Solicited ADR (MedDRA PT)** | **n (%)** | **95% CI** | **n (%)** | **95% CI** | **n (%)** | **95% CI** | **n (%)** | **95% CI** | **n (%)** | **95% CI** | **n (%)** | **95% CI** |
| **At least one solicited ADR** | 202 (56.4) | 51.2-61.5 | 1035 (72.3) | 69.9-74.5 | 124 (50.8) | 44.6 - 57 | 681 (69.8) | 66.8 - 72.6 | 78 (69) | 60 - 76.8 | 350 (77.4) | 73.4 - 81 |
| **Local solicited ADR** |  |  |  |  |  |  |  |  |  |  |  |  |
| Erythema | 16 (4.5) | 2.8-7.1 | 96 (6.7) | 5.5 - 8.1 | 8 (3.3) | 1.7 - 6.3 | 60 (6.1) | 4.8 - 7.8 | 8 (7.1) | 3.6 - 13.4 | 36 (8.0) | 5.8 - 10.8 |
| Haematoma | 9 (2.5) | 1.3-4.7 | 45 (3.1) | 2.4 - 4.2 | 5 (2.0) | 0.7 - 4.7 | 30 (3.1) | 2.2 - 4.4 | 4 (3.5) | 1 - 8.8 | 15 (3.3) | 2 - 5.4 |
| Induration | 1 (0.3) | 1.5-5.1 | 10 (0.7) | 0.4 - 1.3 | 0 (0) | 0 - 1.5 | 7 (0.7) | 0.3 - 1.5 | 1 (0.9) | 0 - 4.8 | 3 (0.7) | 0.1 - 1.9 |
| Inflammation | 48 (13.4) | 10.3-17.3 | 248 (17.3) | 15.4 - 19.4 | 23 (9.4) | 6.4 - 13.7 | 141 (14.4) | 12.4 - 16.8 | 25 (22.1) | 15.5 - 30.6 | 106 (23.5) | 19.8 - 27.6 |
| Pain | 139 (38.8) | 33.9-44.0 | 687 (48.0) | 45.4 - 50.6 | 86 (35.2) | 29.5 - 41.4 | 436 (44.7) | 41.6 - 47.8 | 53 (46.9) | 38 - 56.1 | 249 (55.1) | 50.5 - 59.6 |
| Pruritus | 10 (2.8) | 1.7-5.4 | 66 (4.6) | 3.6 - 5.8 | 5 (2.0) | 0.7 - 4.7 | 46 (4.7) | 3.6 - 6.2 | 5 (4.4) | 1.5 - 10 | 19 (4.2) | 2.7 - 6.5 |
| Injection site reaction* | 0 (0) | 0.0-1.0 | 2 (0.1) | 0 - 0.5 | 0 (0) | 0 - 1.5 | 2 (0.2) | 0 - 0.7 | 0 (0) | 0 - 3.2 | 0 (0) | 0 - 0.8 |
| Swelling | 36 (10.1) | 12.2-19.8 | 280 (19.6) | 17.6 - 21.7 | 22 (9) | 6 - 13.3 | 168 (17.2) | 15 - 19.7 | 14 (12.4) | 7.5 - 19.7 | 111 (24.6) | 20.8 - 28.7 |
| Site warmth | 27 (7.5) | 5.2-10.8 | 101 (7.1) | 5.8 - 8.5 | 13 (5.3) | 3.1 - 8.9 | 57 (5.8) | 4.5 - 7.5 | 14 (12.4) | 7.5 - 19.7 | 43 (9.5) | 7.1 - 12.6 |
| **Systemic solicited ADR** |  |  |  |  |  |  |  |  |  |  |  |  |
| Arthralgia | 39 (10.9) | 8.1-14.5 | 39 (10.9) | 2 - 3.7 | 21 (8.6) | 5.7 - 12.8 | 147 (15.1) | 13 - 17.4 | 18 (15.9) | 10.3 - 23.8 | 75 (16.6) | 13.4 - 20.3 |
| Chills | 39 (10.9) | 8.1-14.5 | 39 (10.9) | 2 - 3.7 | 17 (7) | 4.4 - 10.9 | 188 (19.3) | 16.9 - 21.9 | 22 (19.5) | 13.2 - 27.7 | 145 (32.1) | 27.9 - 36.5 |
| Fatigue | 105 (29.3) | 24.9-34.2 | 105 (29.3) | 6.1 - 8.8 | 56 (23) | 18.1 - 28.6 | 378 (38.7) | 35.7 - 41.8 | 49 (43.4) | 34.6 - 52.6 | 227 (50.2) | 45.6 - 54.8 |
| Headache | 84 (23.5) | 19.4-28.1 | 84 (23.5) | 4.8 - 7.2 | 45 (18.4) | 14.1 - 23.8 | 303 (31.0) | 28.2 - 34 | 39 (34.5) | 26.4 - 43.7 | 191 (42.3) | 37.8 - 46.9 |
| Malaise | 73 (20.4) | 16.5-24.9 | 73 (20.4) | 4.1 - 6.4 | 35 (14.3) | 10.5 - 19.3 | 265 (27.2) | 24.5 - 30 | 38 (33.6) | 25.6 - 42.7 | 150 (33.2) | 29 - 37.7 |
| Myalgia | 65 (18.2) | 14.5-22.5 | 65 (18.2) | 3.6 - 5.7 | 36 (14.8) | 10.9 - 19.7 | 283 (29.0) | 26.2 - 31.9 | 29 (25.7) | 18.5 - 34.4 | 176 (38.9) | 34.6 - 43.5 |
| Nausea | 38 (10.6) | 7.8-14.2 | 38 (10.6) | 1.9 - 3.6 | 24 (9.8) | 6.7 - 14.2 | 98 (10.0) | 8.3 - 12.1 | 14 (12.4) | 7.5 - 19.7 | 70 (15.5) | 12.4 - 19.1 |
| Increased temperature^+^ | 14 (3.9) | 2.3-6.5 | 14 (3.9) | 0.6 - 1.6 | 10 (4.1) | 2.2 - 7.4 | 68 (7.0) | 5.5 - 8.7 | 4 (3.5) | 1 - 8.8 | 36 (8.0) | 5.8 - 10.8 |
| Pyrexia^++^ | 18 (5) | 3.2-7.8 | 18 (5.0) | 0.8 - 2 | 7 (2.9) | 1.4 - 5.8 | 93 (9.5) | 7.8 - 11.5 | 11 (9.7) | 5.5 - 16.6 | 87 (19.2) | 15.9 - 23.1 |
| Hyperpyrexia^+++^ | 0 (0) | 0.0-1.0 | 0 (0) | 0 -1.0 | 0 (0) | 0 - 1.5 | 0 (0) | 0 - 0.4 | 0 (0) | 0 - 3.2 | 0 (0) | 0 - 0.8 |

Abbreviations: ADR = adverse drug reaction; MedDRA=Medical Dictionary for Regulatory Activities; PT=preferred term

ADRs are displayed in absolute counts (percentages).

^*^ Injection site reaction is defined as 2 or more of the following adverse reactions (redness, warmth, pain, swelling) going past the elbow or shoulder.

^+^ Body temperature increased is defined as body temperature between 37.5 and 37.9 degrees Celsius.

^++^ Pyrexia is defined as body temperature between 38.0 and 40.4 degrees Celsius.

^+++^ Hyperpyrexia as body temperature at 40.5 and 42.0 degrees Celsius.

#

# **Supplementary table 3:** List of unsolicited adverse drug reactions between pregnant women and matched non-pregnant women after booster injection, overall and stratified by vaccine brand.

| **COVID-19 vaccine brand** | **All vaccines** |  |  |  | **Pfizer/**  **BioNTech** |  |  |  | **Moderna** |  |  |  |
| --- | --- | --- | --- | --- | --- | --- | --- | --- | --- | --- | --- | --- |
|  | **Cohort**  **N=358** | **95% CI** | **Matched**  **N=1,432** | **95% CI** | **Cohort**  **N=244** | **95% CI** | **Matched**  **N=976** | **95% CI** | **Cohort**  **N=113** | **95% CI** | **Matched**  **N=452** | **95% CI** |
| **Unsolicited ADR (MedDRA PT)** | **n (%)** |  | **n (%)** |  | **n (%)** |  | **n (%)** |  | **n (%)** |  | **n (%)** |  |
| **At least one unsolicited ADR** | **28 (7.8)** | **5.5 - 11.1** | **356 (24.9)** | **22.7 - 27.2** | **18 (7.4)** | **4.7 - 11.4** | **243 (24.9)** | **22.3 - 27.7** | **10 (8.8)** | **4.9 - 15.5** | **113 (25.0)** | **21.2 - 29.2** |
| Abdominal discomfort | 0 (0) | 0 - 1 | 1 (0.1) | 0 - 0.4 | 0 (0) | 0 - 1.5 | 1 (0.1) | 0 - 0.6 | 0 (0) | 0 - 3.2 | 0 (0) | 0 - 0.8 |
| Abdominal distension | 0 (0) | 0 - 1 | 1 (0.1) | 0 - 0.4 | 0 (0) | 0 - 1.5 | 0 (0) | 0 - 0.4 | 0 (0) | 0 - 3.2 | 1 (0.2) | 0 - 1.2 |
| Abdominal pain | 0 (0) | 0 - 1 | 7 (0.5) | 0.2 - 1 | 0 (0) | 0 - 1.5 | 3 (0.3) | 0.1 - 0.9 | 0 (0) | 0 - 3.2 | 4 (8.8) | 0.2 - 2.3 |
| Abdominal pain lower | 0 (0) | 0 - 1 | 1 (0.1) | 0 - 0.4 | 0 (0) | 0 - 1.5 | 0 (0) | 0 - 0.4 | 0 (0) | 0 - 3.2 | 1 (0.2) | 0 - 1.2 |
| Abdominal pain upper | 0 (0) | 0 - 1 | 2 (0.1) | 0 - 0.5 | 0 (0) | 0 - 1.5 | 0 (0) | 0 - 0.4 | 0 (0) | 0 - 3.2 | 2 (0.4) | 0.1 - 1.6 |
| Abnormal dreams | 0 (0) | 0 - 1 | 1 (0.1) | 0 - 0.4 | 0 (0) | 0 - 1.5 | 1 (0.1) | 0 - 0.6 | 0 (0) | 0 - 3.2 | 0 (0) | 0 - 0.8 |
| Abnormal sensation in eye | 0 (0) | 0 - 1 | 1 (0.1) | 0 - 0.4 | 0 (0) | 0 - 1.5 | 0 (0) | 0 - 0.4 | 0 (0) | 0 - 3.2 | 1 (0.2) | 0 - 1.2 |
| Adnexa uteri pain | 0 (0) | 0 - 1 | 1 (0.1) | 0 - 0.4 | 0 (0) | 0 - 1.5 | 1 (0.1) | 0 - 0.6 | 0 (0) | 0 - 3.2 | 0 (0) | 0 - 0.8 |
| Ageusia | 1 (0.3) | 0 - 1.5 | 0 (0) | 0 - 0.3 | 1 (0.4) | 0 - 2.3 | 0 (0) | 0 - 0.4 | 0 (0) | 0 - 3.2 | 0 (0) | 0 - 0.8 |
| Anosmia | 1 (0.3) | 0 - 1.5 | 0 (0) | 0 - 0.3 | 1 (0.4) | 0 - 2.3 | 0 (0) | 0 - 0.4 | 0 (0) | 0 - 3.2 | 0 (0) | 0 - 0.8 |
| Aphthous ulcer | 0 (0) | 0 - 1 | 1 (0.1) | 0 - 0.4 | 0 (0) | 0 - 1.5 | 1 (0.1) | 0 - 0.6 | 0 (0) | 0 - 3.2 | 0 (0) | 0 - 0.8 |
| *Arrhythmia* | *0 (0)* | 0 - 1 | *3 (0.2)* | *0 - 0.6* | *0 (0)* | *0 - 1.5* | *2 (0.2)* | *0 - 0.7* | *0 (0)* | 0 - 3.2 | 1 (0.2) | 0 - 1.2 |
| Asthma | 0 (0) | 0 - 1 | 1 (0.1) | 0 - 0.4 | 0 (0) | 0 - 1.5 | 1 (0.1) | 0 - 0.6 | 0 (0) | 0 - 3.2 | 0 (0) | 0 - 0.8 |
| Axillary pain | 3 (0.8) | 0.2 - 2.4 | 26 (1.8) | 1.2 - 2.6 | 3 (1.2) | 0.3 - 3.6 | 23 (2.6) | 1.6 - 3.5 | 0 (0) | 0 - 3.2 | 3 (0.7) | 0.1 - 1.9 |
| Back pain | 0 (0) | 0 - 1 | 3 (0.2) | 0 - 0.6 | 0 (0) | 0 - 1.5 | 2 (0.2) | 0 - 0.7 | 0 (0) | 0 - 3.2 | 1 (0.2) | 0 - 1.2 |
| Bedridden | 0 (0) | 0 - 1 | 1 (0.1) | 0 - 0.4 | 0 (0) | 0 - 1.5 | 0 (0) | 0 - 0.4 | 0 (0) | 0 - 3.2 | 1 (0.2) | 0 - 1.2 |
| Blood pressure decreased | 0 (0) | 0 - 1 | 1 (0.1) | 0 - 0.4 | 0 (0) | 0 - 1.5 | 1 (0.1) | 0 - 0.6 | 0 (0) | 0 - 3.2 | 0 (0) | 0 - 0.8 |
| Blood pressure increased | 0 (0) | 0 - 1 | 1 (0.1) | 0 - 0.4 | 0 (0) | 0 - 1.5 | 1 (0.1) | 0 - 0.6 | 0 (0) | 0 - 3.2 | 0 (0) | 0 - 0.8 |
| Bone pain | 0 (0) | 0 - 1 | 1 (0.1) | 0 - 0.4 | 0 (0) | 0 - 1.5 | 1 (0.1) | 0 - 0.6 | 0 (0) | 0 - 3.2 | 0 (0) | 0 - 0.8 |
| Breast engorgement | 0 (0) | 0 - 1 | 1 (0.1) | 0 - 0.4 | 0 (0) | 0 - 1.5 | 0 (0) | 0 - 0.4 | 0 (0) | 0 - 3.2 | 1 (0.2) | 0 - 1.2 |
| Breast pain | 0 (0) | 0 - 1 | 3 (0.2) |  | 0 (0) | 0 - 1.5 | *2 (0.2)* |  | 0 (0) | 0 - 3.2 | 1 (0.2) | 0 - 1.2 |
| Bronchial hyperreactivity | 0 (0) | 0 - 1 | 1 (0.1) | 0 - 0.4 | 0 (0) | 0 - 1.5 | 0 (0) | 0 - 0.4 | 0 (0) | 0 - 3.2 | 1 (0.2) | 0 - 1.2 |
| Bronchial irritation | 0 (0) | 0 - 1 | 1 (0.1) | 0 - 0.4 | 0 (0) | 0 - 1.5 | 0 (0) | 0 - 0.4 | 0 (0) | 0 - 3.2 | 1 (0.2) | 0 - 1.2 |
| Bronchitis | 0 (0) | 0 - 1 | 1 (0.1) | 0 - 0.4 | 0 (0) | 0 - 1.5 | 1 (0.1) | 0 - 0.6 | 0 (0) | 0 - 3.2 | 0 (0) | 0 - 0.8 |
| Cardiomyopathy | 0 (0) | 0 - 1 | 1 (0.1) | 0 - 0.4 | 0 (0) | 0 - 1.5 | 0 (0) | 0 - 0.4 | 0 (0) | 0 - 3.2 | 1 (0.2) | 0 - 1.2 |
| Chest discomfort | 0 (0) | 0 - 1 | 3 (0.2) | 0 - 0.6 | 0 (0) | 0 - 1.5 | 1 (0.1) | 0 - 0.6 | 0 (0) | 0 - 3.2 | 2 (0.4) | 0.1 - 1.6 |
| Chest pain | 0 (0) | 0 - 1 | 10 (0.7) | 0.4 - 1.3 | 0 (0) | 0 - 1.5 | 7 (0.7) | 0.3 - 1.5 | 0 (0) | 0 - 3.2 | 3 (0.7) | 0.1 - 1.9 |
| Cold sweat | 0 (0) | 0 - 1 | 1 (0.1) | 0 - 0.4 | 0 (0) | 0 - 1.5 | 1 (0.1) | 0 - 0.6 | 0 (0) | 0 - 3.2 | 0 (0) | 0 - 0.8 |
| **Congenital anomaly** | **1 (0.3)** | **0 - 1.5** | **0 (0)** | **0 - 0.3** | **1 (0.4)** | **0 - 2.3** | **0 (0)** | **0 - 0.4** | **0 (0)** | 0 - 3.2 | **0 (0)** | 0 - 0.8 |
| Conjunctivitis | 0 (0) | 0 - 1 | 1 (0.1) | 0 - 0.4 | 0 (0) | 0 - 1.5 | 1 (0.1) | 0 - 0.6 | 0 (0) | 0 - 3.2 | 0 (0) | 0 - 0.8 |
| Cough | 0 (0) | 0 - 1 | 9 (0.6) | 0.3 - 1.2 | 0 (0) | 0 - 1.5 | 8 (0.8) | 0.4 - 1.6 | 0 (0) | 0 - 3.2 | 1 (0.2) | 0 - 1.2 |
| *COVID-19* | *0 (0)* | 0 - 1 | *2 (0.1)* | *0 - 0.5* | *0 (0)* | *0 - 1.5* | *2 (0.2)* | *0 - 0.7* | *0 (0)* | 0 - 3.2 | *0 (0)* | 0 - 0.8 |
| Dark circles under eyes | 0 (0) | 0 - 1 | 1 (0.1) | 0 - 0.4 | 0 (0) | 0 - 1.5 | 1 (0.1) | 0 - 0.6 | 0 (0) | 0 - 3.2 | 0 (0) | 0 - 0.8 |
| Decreased appetite | 0 (0) | 0 - 1 | 2 (0.1) | 0 - 0.5 | 0 (0) | 0 - 1.5 | 1 (0.1) | 0 - 0.6 | 0 (0) | 0 - 3.2 | 1 (0.2) | 0 - 1.2 |
| Depressed mood | 0 (0) | 0 - 1 | 1 (0.1) | 0 - 0.4 | 0 (0) | 0 - 1.5 | 1 (0.1) | 0 - 0.6 | 0 (0) | 0 - 3.2 | 0 (0) | 0 - 0.8 |
| Diarrhoea | 2 (0.6) | 0.1 - 2 | 8 (0.6) | 0.3 - 1.1 | 2 (0.8) | 0.1 - 2.9 | *2 (0.2)* | 0 - 0.7 | 0 (0) | 0 - 3.2 | 6 (1.3) | 0.6 - 2.9 |
| Dizziness | 1 (0.3) | 0 - 1.5 | 9 (0.6) | 0.3 - 1.2 | 1 (0.4) | 0 - 2.3 | 7 (0.7) | 0.3 - 1.5 | 0 (0) | 0 - 3.2 | 2 (0.4) | 0.1 - 1.6 |
| Dry throat | 0 (0) | 0 - 1 | 1 (0.1) | 0 - 0.4 | 0 (0) | 0 - 1.5 | 1 (0.1) | 0 - 0.6 | 0 (0) | 0 - 3.2 | 0 (0) | 0 - 0.8 |
| Dysmenorrhoea | 0 (0) | 0 - 1 | 1 (0.1) | 0 - 0.4 | 0 (0) | 0 - 1.5 | 0 (0) | 0 - 0.4 | 0 (0) | 0 - 3.2 | 1 (0.2) | 0 - 1.2 |
| Dyspepsia | 0 (0) | 0 - 1 | 1 (0.1) | 0 - 0.4 | 0 (0) | 0 - 1.5 | 0 (0) | 0 - 0.4 | 0 (0) | 0 - 3.2 | 1 (0.2) | 0 - 1.2 |
| Dysphonia | 0 (0) | 0 - 1 | 3 (0.2) | 0 - 0.6 | 0 (0) | 0 - 1.5 | 3 (0.3) | 0.1 - 0.9 | 0 (0) | 0 - 3.2 | 0 (0) | 0 - 0.8 |
| Dyspnoea | 0 (0) | 0 - 1 | 11 (0.8) | 0.4 - 1.4 | 0 (0) | 0 - 1.5 | 8 (0.8) | 0.4 - 1.6 | 0 (0) | 0 - 3.2 | 3 (0.7) | 0.1 - 1.9 |
| Dysstasia | 0 (0) | 0 - 1 | 1 (0.1) | 0 - 0.4 | 0 (0) | 0 - 1.5 | 0 (0) | 0 - 0.4 | 0 (0) | 0 - 3.2 | 1 (0.2) | 0 - 1.2 |
| Ear discomfort | 0 (0) | 0 - 1 | 1 (0.1) | 0 - 0.4 | 0 (0) | 0 - 1.5 | 0 (0) | 0 - 0.4 | 0 (0) | 0 - 3.2 | 0 (0) | 0 - 0.8 |
| Ear pain | 0 (0) | 0 - 1 | 1 (0.1) | 0 - 0.4 | 0 (0) | 0 - 1.5 | 0 (0) | 0 - 0.4 | 0 (0) | 0 - 3.2 | 0 (0) | 0 - 0.8 |
| Eczema | 0 (0) | 0 - 1 | 2 (0.1) | 0 - 0.5 | 0 (0) | 0 - 1.5 | 1 (0.1) | 0 - 0.6 | 0 (0) | 0 - 3.2 | 1 (0.2) | 0 - 1.2 |
| Endometriosis | 0 (0) | 0 - 1 | 1 (0.1) | 0 - 0.4 | 0 (0) | 0 - 1.5 | 1 (0.1) | 0 - 0.6 | 0 (0) | 0 - 3.2 | 0 (0) | 0 - 0.8 |
| Erythema | 1 (0.3) | 0 - 1.5 | 1 (0.1) | 0 - 0.4 | 1 (0.4) | 0 - 2.3 | 1 (0.1) | 0 - 0.6 | 0 (0) | 0 - 3.2 | 0 (0) | 0 - 0.8 |
| Eye pain | 0 (0) | 0 - 1 | 1 (0.1) | 0 - 0.4 | 0 (0) | 0 - 1.5 | 1 (0.1) | 0 - 0.6 | 0 (0) | 0 - 3.2 | 0 (0) | 0 - 0.8 |
| Feeling hot | 0 (0) | 0 - 1 | 1 (0.1) | 0 - 0.4 | 0 (0) | 0 - 1.5 | 1 (0.1) | 0 - 0.6 | 0 (0) | 0 - 3.2 | 0 (0) | 0 - 0.8 |
| **Gastritis** | **0 (0)** | 0 - 1 | **1 (0.1)** | **0 - 0.4** | **0 (0)** | 0 - 1.5 | 1 (0.1) | 0 - 0.6 | **0 (0)** | 0 - 3.2 | **0 (0)** | 0 - 0.8 |
| Gastrointestinal disorder | 0 (0) | 0 - 1 | 3 (0.2) | 0 - 0.6 | 0 (0) | 0 - 1.5 | 1 (0.1) | 0 - 0.6 | 0 (0) | 0 - 3.2 | 2 (0.4) | 0.1 - 1.6 |
| Gastrooesophageal reflux disease | 0 (0) | 0 - 1 | 1 (0.1) | 0 - 0.4 | 0 (0) | 0 - 1.5 | 1 (0.1) | 0 - 0.6 | 0 (0) | 0 - 3.2 | 0 (0) | 0 - 0.8 |
| Genital rash | 0 (0) | 0 - 1 | 1 (0.1) | 0 - 0.4 | 0 (0) | 0 - 1.5 | 1 (0.1) | 0 - 0.6 | 0 (0) | 0 - 3.2 | 0 (0) | 0 - 0.8 |
| Goitre | 0 (0) | 0 - 1 | 1 (0.1) | 0 - 0.4 | 0 (0) | 0 - 1.5 | 1 (0.1) | 0 - 0.6 | 0 (0) | 0 - 3.2 | 0 (0) | 0 - 0.8 |
| **Haemorrhage in pregnancy** | **1 (0.3)** | **0 - 1.5** | **0 (0)** | 0 - 0.3 | 1 (0.4) | **0 - 2.3** | **0 (0)** | **0 - 0.4** | **0 (0)** | 0 - 3.2 | **0 (0)** | 0 - 0.8 |
| Haematoma | 0 (0) | 0 - 1 | 1 (0.1) | 0 - 0.4 | 0 (0) | 0 - 1.5 | 0 (0) | 0 - 0.4 | 0 (0) | 0 - 3.2 | 1 (0.2) | 0 - 1.2 |
| Heart rate decreased | 0 (0) | 0 - 1 | 1 (0.1) | 0 - 0.4 | 0 (0) | 0 - 1.5 | 1 (0.1) | 0 - 0.6 | 0 (0) | 0 - 3.2 | 0 (0) | 0 - 0.8 |
| Heavy menstrual bleeding | 0 (0) | 0 - 1 | 2 (0.1) | 0 - 0.5 | 0 (0) | 0 - 1.5 | *2 (0.2)* | 0 - 0.7 | 0 (0) | 0 - 3.2 | 0 (0) | 0 - 0.8 |
| Herpes simplex | 0 (0) | 0 - 1 | 1 (0.1) | 0 - 0.4 | 0 (0) | 0 - 1.5 | 0 (0) | 0 - 0.4 | 0 (0) | 0 - 3.2 | 1 (0.2) | 0 - 1.2 |
| Hormone level abnormal | 0 (0) | 0 - 1 | 2 (0.1) | 0 - 0.5 | 0 (0) | 0 - 1.5 | *2 (0.2)* | 0 - 0.7 | 0 (0) | 0 - 3.2 | 0 (0) | 0 - 0.8 |
| Hot flush | 0 (0) | 0 - 1 | 2 (0.1) | 0 - 0.5 | 0 (0) | 0 - 1.5 | 0 (0) | 0 - 0.4 | 0 (0) | 0 - 3.2 | 2 (0.4) | 0.1 - 1.6 |
| *Hypersomnia* | *0 (0)* | 0 - 1 | *1 (0.1)* | *0 - 0.4* | *0 (0)* | *0 - 1.5* | 1 (0.1) | 0 - 0.6 | *0 (0)* | 0 - 3.2 | *0 (0)* | 0 - 0.8 |
| Hypertension | 0 (0) | 0 - 1 | 2 (0.1) | 0 - 0.5 | 0 (0) | 0 - 1.5 | 1 (0.1) | 0 - 0.6 | 0 (0) | 0 - 3.2 | 0 (0) | 0 - 0.8 |
| Hypoaesthesia | 1 (0.3) | 0 - 1.5 | 1 (0.1) | 0 - 0.4 | 0 (0) | 0 - 1.5 | 1 (0.1) | 0 - 0.6 | 1 (0.9) | 0 - 4.8 | 0 (0) | 0 - 0.8 |
| Hypokinesia | 1 (0.3) | 0 - 1.5 | 0 (0) |  | 1 (0.4) | 0 - 2.3 | 0 (0) | 0 - 0.4 | 0 (0) | 0 - 3.2 | 0 (0) | 0 - 0.8 |
| Influenza | 1 (0.3) | 0 - 1.5 | 2 (0.1) | 0 - 0.5 | 0 (0) | 0 - 1.5 | 1 (0.1) | 0 - 0.6 | 1 (0.9) | 0 - 4.8 | 1 (0.2) | 0 - 1.2 |
| Injected limb mobility decreased | 0 (0) | 0 - 1 | 2 (0.1) | 0 - 0.5 | 0 (0) | 0 - 1.5 | 1 (0.1) | 0 - 0.6 | 0 (0) | 0 - 3.2 | 0 (0) | 0 - 0.8 |
| Injection site joint inflammation | 0 (0) | 0 - 1 | 1 (0.1) | 0 - 0.4 | 0 (0) | 0 - 1.5 | 1 (0.1) | 0 - 0.6 | 0 (0) | 0 - 3.2 | 0 (0) | 0 - 0.8 |
| Injection site rash | 0 (0) | 0 - 1 | 1 (0.1) | 0 - 0.4 | 0 (0) | 0 - 1.5 | 1 (0.1) | 0 - 0.6 | 0 (0) | 0 - 3.2 | 0 (0) | 0 - 0.8 |
| Injection site reaction | 0 (0) | 0 - 1 | 2 (0.1) | 0 - 0.5 | 0 (0) | 0 - 1.5 | 1 (0.1) | 0 - 0.6 | 0 (0) | 0 - 3.2 | 0 (0) | 0 - 0.8 |
| Insomnia | 2 (0.6) | 0.1 - 2 | 3 (0.2) | 0 - 0.6 | 0 (0) | 0 - 1.5 | 3 (0.3) |  | 0 (0) | 0 - 3.2 | 0 (0) | 0 - 0.8 |
| Joint swelling | 0 (0) | 0 - 1 | 1 (0.1) | 0 - 0.4 | 0 (0) | 0 - 1.5 | 1 (0.1) | 0 - 0.6 | 0 (0) | 0 - 3.2 | 0 (0) | 0 - 0.8 |
| Limb discomfort | 0 (0) | 0 - 1 | 2 (0.1) | 0 - 0.5 | 0 (0) | 0 - 1.5 | *2 (0.2)* | 0 - 0.7 | 0 (0) | 0 - 3.2 | 0 (0) | 0 - 0.8 |
| **Loss of consciousness** | **1 (0.3)** | **0 - 1.5** | **0 (0)** | 0 - 0.3 | 1 (0.4) | **0 - 2.3** | **0 (0)** | **0 - 0.4** | **0 (0)** | 0 - 3.2 | **0 (0)** | 0 - 0.8 |
| Lymphadenitis | 1 (0.3) | 0 - 1.5 | 60 (4.2) | 3.3 - 5.4 | 0 (0) | 0 - 1.5 | 35 (3.6) | 2.6 - 4.9 | 1 (0.9) | 0 - 4.8 | 25 (5.5) | 3.8 - 8 |
| Lymphadenopathy | 6 (1.7) | 0.8 - 3.6 | 57 (4.0) | 3.1 - 5.1 | 4 (1.6) | 0.4 - 4.1 | 50 (5.1) | 3.9 - 6.7 | 2 (1.8) | 0.2 - 6.2 | 7 (3.3) | 0.8 - 3.2 |
| Mastitis | 0 (0) | 0 - 1 | 1 (0.1) | 0 - 0.4 | 0 (0) | 0 - 1.5 | 0 (0) | 0 - 0.4 | 0 (0) | 0 - 3.2 | 1 (0.2) | 0 - 1.2 |
| Menstrual disorder | 0 (0) | 0 - 1 | 48 (3.4) | 2.5 - 4.4 | 0 (0) | 0 - 1.5 | 33 (3.4) | 2.4 - 4.7 | 0 (0) | 0 - 3.2 | 15 (3.3) | 2 - 5.4 |
| Menstruation delayed | 0 (0) | 0 - 1 | 5 (0.3) | 0.1 - 0.8 | 0 (0) | 0 - 1.5 | 5 (0.5) | 0.2 - 1.2 | 0 (0) | 0 - 3.2 | 0 (0) | 0 - 0.8 |
| Menstruation irregular | 0 (0) | 0 - 1 | 7 (0.5) | 0.2 - 1 | 0 (0) | 0 - 1.5 | 4 (0.4) | 0.1 - 1 | 0 (0) | 0 - 3.2 | 3 (0.7) | 0.1 - 1.9 |
| Muscle contractions involuntary | 0 (0) | 0 - 1 | 1 (0.1) | 0 - 0.4 | 0 (0) | 0 - 1.5 | 0 (0) | 0 - 0.4 | 0 (0) | 0 - 3.2 | 1 (0.2) | 0 - 1.2 |
| Muscular weakness | 1 (0.3) | 0 - 1.5 | 0 (0) | 0 - 0.3 | 1 (0.4) | 0 - 2.3 | 0 (0) | 0 - 0.4 | 0 (0) | 0 - 3.2 | 0 (0) | 0 - 0.8 |
| Musculoskeletal chest pain | 0 (0) | 0 - 1 | 1 (0.1) | 0 - 0.4 | 0 (0) | 0 - 1.5 | 0 (0) | 0 - 0.4 | 0 (0) | 0 - 3.2 | 1 (0.2) | 0 - 1.2 |
| Musculoskeletal stiffness | 0 (0) | 0 - 1 | 4 (0.3) | 0.1 - 0.7 | 0 (0) | 0 - 1.5 | 3 (0.3) | 0.1 - 0.9 | 0 (0) | 0 - 3.2 | 1 (0.2) | 0 - 1.2 |
| Nasal dryness | 0 (0) | 0 - 1 | 1 (0.1) | 0 - 0.4 | 0 (0) | 0 - 1.5 | 1 (0.1) | 0 - 0.6 | 0 (0) | 0 - 3.2 | 0 (0) | 0 - 0.8 |
| Nasopharyngitis | 3 (0.8) | 0.2 - 2.4 | 7 (0.5) | 0.2 - 1 | 1 (0.4) | 0 - 2.3 | 5 (0.5) | 0.2 - 1.2 | 2 (1.8) | 0.2 - 6.2 | 2 (0.4) | 0.1 - 1.6 |
| Neck pain | 0 (0) | 0 - 1 | 3 (0.2) | 0 - 0.6 | 0 (0) | 0 - 1.5 | *2 (0.2)* | 0 - 0.7 | 0 (0) | 0 - 3.2 | 1 (0.2) | 0 - 1.2 |
| Nodule | 0 (0) | 0 - 1 | 1 (0.1) | 0 - 0.4 | 0 (0) | 0 - 1.5 | 0 (0) | 0 - 0.4 | 0 (0) | 0 - 3.2 | 1 (0.2) | 0 - 1.2 |
| Oligomenorrhoea | 0 (0) | 0 - 1 | 1 (0.1) | 0 - 0.4 | 0 (0) | 0 - 1.5 | 1 (0.1) | 0 - 0.6 | 0 (0) | 0 - 3.2 | 0 (0) | 0 - 0.8 |
| Oral discomfort | 0 (0) | 0 - 1 | 1 (0.1) | 0 - 0.4 | 0 (0) | 0 - 1.5 | 1 (0.1) | 0 - 0.6 | 0 (0) | 0 - 3.2 | 1 (0.2) | 0 - 1.2 |
| Oral herpes | 0 (0) | 0 - 1 | 1 (0.1) | 0 - 0.4 | 0 (0) | 0 - 1.5 | 1 (0.1) | 0 - 0.6 | 0 (0) | 0 - 3.2 | 0 (0) | 0 - 0.8 |
| Oropharyngeal discomfort | 1 (0.3) | 0 - 1.5 | 0 (0) | 0 - 0.3 | 0 (0) | 0 - 1.5 | 0 (0) | 0 - 0.4 | 1 (0.9) | 0 - 4.8 | 0 (0) | 0 - 0.8 |
| Oropharyngeal pain | 2 (0.6) | 0.1 - 2 | 8 (0.6) | 0.3 - 1.1 | 1 (0.4) | 0 - 2.3 | 5 (0.5) | 0.2 - 1.2 | 1 (0.9) | 0 - 4.8 | 3 (0.7) | 0.1 - 1.9 |
| Pain | 0 (0) | 0 - 1 | 5 (0.3) | 0.1 - 0.8 | 0 (0) | 0 - 1.5 | 4 (0.4) | 0.1 - 1 | 0 (0) | 0 - 3.2 | 1 (0.2) | 0 - 1.2 |
| Pain in extremity | 2 (0.6) | 0.1 - 2 | 11 (0.8) | 0.4 - 1.4 | 1 (0.4) | 0 - 2.3 | 6 (0.6) | 0.3 - 1.3 | 1 (0.9) | 0 - 4.8 | 5 (1.1) | 0.4 - 2.6 |
| Palpitations | 0 (0) | 0 - 1 | 1 (0.1) | 0 - 0.4 | 0 (0) | 0 - 1.5 | 1 (0.1) | 0 - 0.6 | 0 (0) | 0 - 3.2 | 0 (0) | 0 - 0.8 |
| Paraesthesia | 0 (0) | 0 - 1 | 12 (0.8) | 0.5 - 1.5 | 0 (0) | 0 - 1.5 | 6 (0.6) | 0.3 - 1.3 | 0 (0) | 0 - 3.2 | 6 (1.3) | 0.6 - 2.9 |
| Paraesthesia oral | 0 (0) | 0 - 1 | 2 (0.1) | 0 - 0.5 | 0 (0) | 0 - 1.5 | 5 (0.5) | 0.2 - 1.2 | 0 (0) | 0 - 3.2 | 1 (0.2) | 0 - 1.2 |
| Periorbital swelling | 1 (0.3) | 0 - 1.5 | 0 (0) | 0 - 0.3 | 1 (0.4) | 0 - 2.3 | 0 (0) | 0 - 0.4 | 0 (0) | 0 - 3.2 | 0 (0) | 0 - 0.8 |
| Peripheral swelling | 0 (0) | 0 - 1 | 1 (0.1) | 0 - 0.4 | 0 (0) | 0 - 1.5 | 0 (0) | 0 - 0.4 | 0 (0) | 0 - 3.2 | 1 (0.2) | 0 - 1.2 |
| Pharyngitis | 0 (0) | 0 - 1 | 1 (0.1) | 0 - 0.4 | 0 (0) | 0 - 1.5 | 1 (0.1) | 0 - 0.6 | 0 (0) | 0 - 3.2 | 0 (0) | 0 - 0.8 |
| Photophobia | 0 (0) | 0 - 1 | 2 (0.1) | 0 - 0.5 | 0 (0) | 0 - 1.5 | 0 (0) | 0 - 0.4 | 0 (0) | 0 - 3.2 | 2 (0.4) | 0.1 - 1.6 |
| Polymenorrhoea | 0 (0) | 0 - 1 | 1 (0.1) | 0 - 0.4 | 0 (0) | 0 - 1.5 | 0 (0) | 0 - 0.4 | 0 (0) | 0 - 3.2 | 1 (0.2) | 0 - 1.2 |
| Presyncope | 1 (0.3) | 0 - 1.5 | 1 (0.1) | 0 - 0.4 | 1 (0.4) | 0 - 2.3 | 0 (0) | 0 - 0.4 | 0 (0) | 0 - 3.2 | 1 (0.2) | 0 - 1.2 |
| Pruritus | 0 (0) | 0 - 1 | 6 (0.4) | 0.2 - 0.9 | 0 (0) | 0 - 1.5 | 3 (0.3) | 0.1 - 0.9 | 0 (0) | 0 - 3.2 | 3 (0.7) | 0.1 - 1.9 |
| Psoriasis | 0 (0) | 0 - 1 | 1 (0.1) | 0 - 0.4 | 0 (0) | 0 - 1.5 | 4 (0.4) | 0.1 - 1 | 0 (0) | 0 - 3.2 | 1 (0.2) | 0 - 1.2 |
| Rash | 1 (0.3) | 0 - 1.5 | 8 (0.6) | 0.3 - 1.1 | 1 (0.4) | 0 - 2.3 | 0 (0) | 0 - 0.4 | 0 (0) | 0 - 3.2 | 4 (8.8) | 0.2 - 2.3 |
| Rash macular | 0 (0) | 0 - 1 | 2 (0.1) | 0 - 0.5 | 0 (0) | 0 - 1.5 | *2 (0.2)* | 0 - 0.7 | 0 (0) | 0 - 3.2 | 0 (0) | 0 - 0.8 |
| Rhinitis | 0 (0) | 0 - 1 | 3 (0.2) | 0 - 0.6 | 0 (0) | 0 - 1.5 | 3 (0.3) | 0.1 - 0.9 | 0 (0) | 0 - 3.2 | 0 (0) | 0 - 0.8 |
| Rhinorrhoea | 0 (0) | 0 - 1 | 1 (0.1) | 0 - 0.4 | 0 (0) | 0 - 1.5 | 1 (0.1) | 0 - 0.6 | 0 (0) | 0 - 3.2 | 0 (0) | 0 - 0.8 |
| SARS-CoV-2 test positive | 0 (0) | 0 - 1 | 1 (0.1) | 0 - 0.4 | 0 (0) | 0 - 1.5 | 1 (0.1) | 0 - 0.6 | 0 (0) | 0 - 3.2 | 0 (0) | 0 - 0.8 |
| Sciatica | 0 (0) | 0 - 1 | 1 (0.1) | 0 - 0.4 | 0 (0) | 0 - 1.5 | 1 (0.1) | 0 - 0.6 | 0 (0) | 0 - 3.2 | 0 (0) | 0 - 0.8 |
| Sinusitis | 0 (0) | 0 - 1 | 1 (0.1) | 0 - 0.4 | 0 (0) | 0 - 1.5 | 0 (0) | 0 - 0.4 | 0 (0) | 0 - 3.2 | 1 (0.2) | 0 - 1.2 |
| Sleep disorder | 0 (0) | 0 - 1 | 4 (0.3) | 0.1 - 0.7 | 0 (0) | 0 - 1.5 | 3 (0.3) | 0.1 - 0.9 | 0 (0) | 0 - 3.2 | 1 (0.2) | 0 - 1.2 |
| Stomatitis | 0 (0) | 0 - 1 | 1 (0.1) | 0 - 0.4 | 0 (0) | 0 - 1.5 | 1 (0.1) | 0 - 0.6 | 0 (0) | 0 - 3.2 | 0 (0) | 0 - 0.8 |
| Suppressed lactation | 0 (0) | 0 - 1 | 1 (0.1) | 0 - 0.4 | 0 (0) | 0 - 1.5 | 0 (0) | 0 - 0.4 | 0 (0) | 0 - 3.2 | 1 (0.2) | 0 - 1.2 |
| Swelling | 0 (0) | 0 - 1 | 2 (0.1) | 0 - 0.5 | 0 (0) | 0 - 1.5 | 1 (0.1) | 0 - 0.6 | 0 (0) | 0 - 3.2 | 1 (0.2) | 0 - 1.2 |
| Swelling face | 0 (0) | 0 - 1 | 1 (0.1) | 0 - 0.4 | 0 (0) | 0 - 1.5 | 0 (0) | 0 - 0.4 | 0 (0) | 0 - 3.2 | 1 (0.2) | 0 - 1.2 |
| Syncope | 0 (0) | 0 - 1 | 1 (0.1) | 0 - 0.4 | 0 (0) | 0 - 1.5 | 1 (0.1) | 0 - 0.6 | 0 (0) | 0 - 3.2 | 0 (0) | 0 - 0.8 |
| Tachycardia | 0 (0) | 0 - 1 | 7 (0.5) | 0.2 - 1 | 0 (0) | 0 - 1.5 | 5 (0.5) | 0.2 - 1.2 | 0 (0) | 0 - 3.2 | 2 (0.4) | 0.1 - 1.6 |
| Tendonitis | 0 (0) | 0 - 1 | 1 (0.1) | 0 - 0.4 | 0 (0) | 0 - 1.5 | 1 (0.1) | 0 - 0.6 | 0 (0) | 0 - 3.2 | 0 (0) | 0 - 0.8 |
| Tinnitus | 0 (0) | 0 - 1 | 1 (0.1) | 0 - 0.4 | 0 (0) | 0 - 1.5 | 1 (0.1) | 0 - 0.6 | 0 (0) | 0 - 3.2 | 0 (0) | 0 - 0.8 |
| Vaccination site joint swelling | 0 (0) | 0 - 1 | 1 (0.1) | 0 - 0.4 | 0 (0) | 0 - 1.5 | 1 (0.1) | 0 - 0.6 | 0 (0) | 0 - 3.2 | 0 (0) | 0 - 0.8 |
| Varicose vein | 0 (0) | 0 - 1 | 1 (0.1) | 0 - 0.4 | 0 (0) | 0 - 1.5 | 1 (0.1) | 0 - 0.6 | 0 (0) | 0 - 3.2 | 0 (0) | 0 - 0.8 |
| Vertigo | 0 (0) | 0 - 1 | 4 (0.3) | 0.1 - 0.7 | 0 (0) | 0 - 1.5 | 4 (0.4) | 0.1 - 1 | 0 (0) | 0 - 3.2 | 0 (0) | 0 - 0.8 |
| Vision blurred | 0 (0) | 0 - 1 | 1 (0.1) | 0 - 0.4 | 0 (0) | 0 - 1.5 | 1 (0.1) | 0 - 0.6 | 0 (0) | 0 - 3.2 | 0 (0) | 0 - 0.8 |
| Visual acuity reduced | 0 (0) | 0 - 1 | 1 (0.1) | 0 - 0.4 | 0 (0) | 0 - 1.5 | 1 (0.1) | 0 - 0.6 | 0 (0) | 0 - 3.2 | 0 (0) | 0 - 0.8 |
| Vomiting | 2 (0.6) | 0.1 - 2 | 9 (0.6) | 0.3 - 1.2 | 1 (0.4) | 0 - 2.3 | 5 (0.5) | 0.2 - 1.2 | 1 (0.9) | 0 - 4.8 | 4 (8.8) | 0.2 - 2.3 |

Abbreviations: ADR = adverse drug reaction; MedDRA=Medical Dictionary for Regulatory Activities; PT=preferred term

ADRs are displayed in absolute counts (percentages).

Adverse events of special interest (AESI) are in italic. Serious adverse events are in bold.

# **Supplementary table 4:** Adverse drug reactions according to mental health disorders

|  | **No mental disorders**  **N=1717** | **Mental disorders**  **N=73** | **p-value** |
| --- | --- | --- | --- |
| **Solicited ADRs** | **N (%)** | **N (%)** |  |
| Yes | 1116 (65.0) | 59 (80.8) | 0.005 |
| No | 601 (35.0) | 14 (19.2) |  |
| **Unsolicited ADRs** | **N (%)** | **N (%)** |  |
| Yes | 341 (19.9) | 21 (28.8) | 0.06 |
| No | 1376 (80.1) | 52 (71.2) |  |
| **Serious ADRs** | **N (%)** | **N (%)** |  |
| Yes | 8 (0.5) | 0 (0) | 1 |
| No | 1709 (99.5) | 73 (100) |  |

ADRs: Adverse drug reactions.

# **Supplementary table 5:** Characteristics associated with completing the end of pregnancy questionnaire among pregnant women vaccinated with COVID-19 booster.

|  | **Pregnant women who completed end of pregnancy questionnaire**  **N=200** | | **Pregnant women lost to follow-up**  **N=158** | | **p-value** |
| --- | --- | --- | --- | --- | --- |
| **Vaccine brand** | **n** | **%** | **n** | **%** | **p-value** |
| BioNTech Pfizer | 145 | 72.5 | 99 | 62.7 |  |
| Moderna | 55 | 27.5 | 58 | 36.7 | 0.09 |
| Unknown | 0 | 0 | 1 | 0.6 |  |
| **Baseline characteristics** | n | % | n | % | **p-value** |
| 18-24 years | 0 | 0 | 2 | 1.3 |  |
| **Age** 25-29 years | 9 | 4.5 | 17 | 10.8 | 0.03 |
| 30-39 years | 166 | 83.0 | 126 | 79.8 |  |
| 40-49 years | 25 | 12.5 | 13 | 8.2 |  |
| France* | 0 | 0 | 37 | 23.4 |  |
| Ireland | 116 | 58.0 | 47 | 29.7 |  |
| Italy | 27 | 13.5 | 32 | 20.2 |  |
| **Country of residence** Portugal | 6 | 3.0 | 4 | 2.5 |  |
| Romania | 3 | 1.5 | 0 | 0 | 0.0009* |
| Spain | 2 | 1.0 | 1 | 0.6 |  |
| Switzerland | 46 | 23.0 | 35 | 22.1 |  |
| United Kingdom | 0 | 0 | 2 | 1.0 |  |
| **Medical history (MedDRA PT)** | **n** | **%** | **n** | **%** | **p-value** |
| **At least one medical condition** | **26** | **13** | **17** | **10.8** | **0.52** |
| Cardiovascular diseases | 1 | 0.5 | 1 | 0.6 | 0.87 |
| Diabetes mellitus | 2 | 1.0 | 4 | 2.5 | 0.26 |
| Hypertension | 2 | 1.0 | 2 | 1.3 | 0.81 |
| Immunosuppression | 2 | 1.0 | 2 | 1.3 | 0.81 |
| Liver diseases | 1 | 0.5 | 0 | 0 | # |
| Lung diseases | 16 | 8.0 | 5 | 3.2 | 0.05 |
| Mental disorders | 4 | 2.0 | 2 | 1.3 | 0.59 |
| Malignant tumors | 0 | 0 | 0 | 0 | # |
| Neurological diseases | 1 | 0.5 | 1 | 0.6 | 0.87 |
| Renal diseases | 0 | 0 | 1 | 0.6 | # |
| **Adverse outcomes** | **n** | **%** | **n** | **%** | **p-value** |
| Local solicited ADRs | 82 | 41.0 | 65 | 41.1 | 0.98 |
| Systemic solicited ADRs | 91 | 45.5 | 71 | 44.9 | 0.91 |
| Unsolicited ADRs | 14 | 8.9 | 14 | 7.0 | 0.51 |
| AESI | 0 | 0 | 0 | 0 | # |
| Serious ADRs | 2 | 1.0 | 1 | 0.6 | 0.51 |
| COVID-19 after vaccination | 88 | 44.0 | 48 | 30.4 | 0.08 |

ADRs: Adverse drug reactions, AESI: Adverse event of Special Interest

Pregnant lost to follow-up are the women who did not complete the “end of pregnancy” questionnaire sent 45 days after the delivery due date.

*Initially, France did not participate in the study among special populations, such as pregnant women. As pregnant women were not asked to complete the ‘end of pregnancy’ questionnaire in France, there were not included in the chi^2^ test.

# Chi^2^ test was not feasible because of no occurrence in at least one cell.

**Supplementary Table 6**: Adverse drugs reactions (ADR) stratified by the number of comorbidities.

|  | **Pregnant cohort** | | | **Matched non-pregnant cohort** | | | |
| --- | --- | --- | --- | --- | --- | --- | --- |
| **Number of comorbidities** | **0** | **1** | **2** | **0** | **1** | **2** | **≥ 3** |
| N | **315** | **39** | **4** | 1214 | 183 | 29 | 6 |
| **At least one solicited ADR** | | | | | | | |
| Yes | 179 (56.8) | 20 (51.3) | 3 (75.0) | 864 (71.2) | 139 (76.0) | 27 (93.1) | 5 (83.3) |
| **Local solicited ADR (MedDRA PT)** | | | | | | | |
| Erythema | 13 (4.1) | 2 (5.1) | 1 (25) | 79 (6.5) | 15 (8.2) | 2 (6.9) | 0 (0) |
| Haematoma | 8 (2.5) | 1 (2.6) | 0 (0) | 37 (3) | 7 (3.8) | 1 (3.4) | 0 (0) |
| Induration | 1 (0.3) | 0 (0) | 0 (0) | 8 (0.7) | 2 (1.1) | 0 (0) | 0 (0) |
| Inflammation | 40 (12.7) | 7 (17.9) | 1 (25) | 201 (16.6) | 37 (20.2) | 8 (27.6) | 2 (33.3) |
| Pain | 123 (39) | 14 (35.9) | 2 (50) | 564 (46.5) | 97 (53) | 21 (72.4) | 5 (83.3) |
| Pruritus | 9 (2.9) | 1 (2.6) | 0 (0) | 46 (3.8) | 17 (9.3) | 2 (6.9) | 1 (16.7) |
| Local reaction* | 0 (0) | 0 (0) | 0 (0) | 2 (0.2) | 0 (0) | 0 (0) | 0 (0) |
| Swelling | 30 (9.5) | 5 (12.8) | 1 (25) | 230 (18.9) | 40 (21.9) | 9 (31) | 1 (16.7) |
| Warmth | 22 (7) | 4 (10.3) | 1 (25) | 77 (6.3) | 17 (9.3) | 5 (17.2) | 2 (33.3) |
| **Systemic solicited ADR (MedDRA PT)** | | | | | | | |
| Arthralgia | 36 (11.4) | 2 (5.1) | 1 (25) | 179 (14.7) | 34 (18.6) | 9 (31) | 1 (16.7) |
| Chills | 34 (10.8) | 4 (10.3) | 1 (25) | 286 (23.6) | 39 (21.3) | 8 (27.6) | 1 (16.7) |
| Fatigue | 93 (29.5) | 11 (28.2) | 1 (25) | 501 (41.3) | 86 (47) | 18 (62.1) | 2 (33.3) |
| Headache | 75 (23.8) | 8 (20.5) | 1 (25) | 415 (34.2) | 66 (36.1) | 13 (44.8) | 3 (50) |
| Malaise | 65 (20.6) | 7 (17.9) | 1 (25) | 343 (28.3) | 60 (32.8) | 12 (41.4) | 3 (50) |
| Myalgia | 55 (17.5) | 7 (17.9) | 3 (75) | 383 (31.5) | 63 (34.4) | 14 (48.3) | 2 (33.3) |
| Nausea | 33 (10.5) | 5 (12.8) | 0 (0) | 130 (10.7) | 31 (16.9) | 5 (17.2) | 2 (33.3) |
| Body temperature increased+ | 12 (3.8) | 2 (5.1) | 0 (0) | 92 (7.6) | 7 (3.8) | 5 (17.2) | 0 (0) |
| Pyrexia++ | 16 (5.1) | 2 (5.1) | 0 (0) | 151 (12.4) | 24 (13.1) | 4 (13.8) | 2 (33.3) |
| Hyperpyrexia+++ | 0 (0) | 0 (0) | 0 (0) | 0 (0) | 0 (0) | 0 (0) | 0 (0) |

In the pregnant cohort, there were no subjects with three or more comorbidities.

Abbreviations: ADR = adverse drug reaction; MedDRA=Medical Dictionary for Regulatory Activities; PT=preferred term

*Local injection site reaction is defined as 2 or more of the following adverse reactions (redness, warmth, pain, swelling).

^+^ Body temperature increased is defined as body temperature between 37.5 and 37.9 degrees Celsius.

^++^ Pyrexia is defined as body temperature between 38.0 and 40.4 degrees Celsius.

^+++^ Hyperpyrexia as body temperature at 40.5 and 42.0 degrees Celsius.

# **Supplementary Table 7:** Adverse drugs reactions (ADR) stratified by country

|  | **France** | | **Ireland** | | **Italy** | | **Portugal** | | **Romania** | | **Spain** | | **Switzerland** | | | **United Kindom** | |
| --- | --- | --- | --- | --- | --- | --- | --- | --- | --- | --- | --- | --- | --- | --- | --- | --- | --- |
|  | **Pregnant cohort** | **Matched non-pregnant cohort** | **Pregnant cohort** | **Matched non-pregnant cohort** | **Pregnant cohort** | **Matched non-pregnant cohort** | **Pregnant cohort** | **Matched non-pregnant cohort** | **Pregnant cohort** | **Matched non-pregnant cohort** | **Pregnant cohort** | **Matched non-pregnant cohort** | **Pregnant cohort** | **Matched non-pregnant cohort** | **Pregnant cohort** | | **Matched non-pregnant cohort** |
| **N** | **37** | **726** | **163** | **10** | **59** | **414** | **10** | **21** | **3** | **48** | **3** | **54** | **81** | **16** | **2** | | **143** |
| **At least one solicited ADR** | | | | | | | | | | | | | | | | | |
| Yes | 22 (59.5) | 522 (71.9) | 75 (46.0) | 4 (40.0) | 38 (64.4) | 308 (74.4) | 5 (50.0) | 14 (66.7) | 3 (100) | 40 (83.3) | 3 (100) | 43 (79.6) | 55 (67.9) | 11 (68.8) | 1 (50.0) | | 93 (65.0) |
| **Local solicited ADR (MedDRA PT)** | | | | | | | | | | | | | | | | | |
| Erythema | 0 (0.0) | 43 (5.9) | 8 (4.9) | 0 (0) | 1 (1.7) | 30 (7.2) | 0 (0) | 4 (19) | 0 (0) | 5 (10.4) | 0 (0) | 9 (16.7) | 6 (7.4) | 0 (0) | 1 (50) | | 5 (3.5) |
| Haematoma | 2 (5.4) | 26 (3.6) | 2 (1.2) | 0 (0) | 1 (1.7) | 10 (2.4) | 0 (0) | 0 (0) | 0 (0) | 0 (0) | 0 (0) | 1 (1.9) | 4 (4.9) | 2 (12.5) | 0 (0) | | 6 (4.2) |
| Induration | 0 (0.0) | 5 (0.7) | 0 (0) | 0 (0) | 0 (0) | 3 (0.7) | 0 (0) | 0 (0) | 0 (0) | 0 (0) | 0 (0) | 2 (3.7) | 1 (1.2) | 0 (0) | 0 (0) | | 0 (0) |
| Inflammation | 8 (21.6) | 141 (19.4) | 13 (8) | 0 (0) | 4 (6.8) | 64 (15.5) | 1 (10) | 4 (19) | 0 (0) | 8 (16.7) | 0 (0) | 17 (31.5) | 22 (27.2) | 4 (25) | 0 (0) | | 10 (7) |
| Pain | 17 (45.9) | 349 (48.1) | 47 (28.8) | 1 (10) | 26 (44.1) | 206 (49.8) | 3 (30) | 5 (23.8) | 3 (100) | 31 (64.6) | 2 (66.7) | 30 (55.6) | 40 (49.4) | 9 (56.3) | 1 (50) | | 56 (39.2) |
| Pruritus | 0 (0.0) | 29 (4) | 5 (3.1) | 0 (0) | 0 (0) | 21 (5.1) | 0 (0) | 1 (4.8) | 0 (0) | 1 (2.1) | 0 (0) | 8 (14.8) | 4 (4.9) | 1 (6.3) | 1 (50) | | 5 (3.5) |
| Local reaction* | 0 (0.0) | 0 (0) | 0 (0) | 0 (0) | 0 (0) | 2 (0.5) | 0 (0) | 0 (0) | 0 (0) | 0 (0) | 0 (0) | 0 (0) | 0 (0) | 0 (0) | 0 (0) | | 0 (0) |
| Swelling | 6 (16.2) | 155 (21.3) | 10 (6.1) | 0 (0) | 4 (6.8) | 81 (19.6) | 2 (20) | 6 (28.6) | 0 (0) | 5 (10.4) | 0 (0) | 18 (33.3) | 13 (16) | 2 (12.5) | 1 (50) | | 13 (9.1) |
| Warmth | 2 (5.4) | 47 (6.5) | 8 (4.9) | 1 (10) | 2 (3.4) | 22 (5.3) | 1 (10) | 3 (14.3) | 0 (0) | 4 (8.3) | 0 (0) | 8 (14.8) | 14 (17.3) | 2 (12.5) | 0 (0) | | 14 (9.8) |
| **Systemic solicited ADR (MedDRA PT)** | | | | | | | | | | | | | | | | | |
| Arthralgia | 1 (2.7) | 85 (11.7) | 12 (7.4) | 0 (0) | 9 (15.3) | 90 (21.7) | 2 (20) | 2 (9.5) | 1 (33.3) | 6 (12.5) | 3 (100) | 13 (24.1) | 11 (13.6) | 5 (31.3) | 0 (0) | | 22 (15.4) |
| Chills | 8 (21.6) | 153 (21.1) | 14 (8.6) | 1 (10) | 8 (13.6) | 116 (28) | 0 (0) | 4 (19) | 1 (33.3) | 9 (18.8) | 2 (66.7) | 18 (33.3) | 6 (7.4) | 2 (12.5) | 0 (0) | | 31 (21.7) |
| Fatigue | 14 (37.8) | 333 (45.9) | 38 (23.3) | 3 (30) | 17 (28.8) | 170 (41.1) | 1 (10) | 5 (23.8) | 1 (33.3) | 21 (43.8) | 1 (33.3) | 19 (35.2) | 32 (39.5) | 5 (31.3) | 1 (50) | | 51 (35.7) |
| Headache | 8 (21.6) | 246 (33.9) | 30 (18.4) | 1 (10) | 11 (18.6) | 154 (37.2) | 2 (20) | 6 (28.6) | 1 (33.3) | 13 (27.1) | 1 (33.3) | 23 (42.6) | 30 (37) | 6 (37.5) | 1 (50) | | 48 (33.6) |
| Malaise | 8 (21.6) | 186 (25.6) | 31 (19) | 0 (0) | 12 (20.3) | 139 (33.6) | 1 (10) | 6 (28.6) | 1 (33.3) | 9 (18.8) | 1 (33.3) | 25 (46.3) | 19 (23.5) | 4 (25) | 0 (0) | | 49 (34.3) |
| Myalgia | 10 (27.0) | 222 (30.6) | 24 (14.7) | 2 (20) | 12 (20.3) | 142 (34.3) | 3 (30) | 8 (38.1) | 1 (33.3) | 13 (27.1) | 2 (66.7) | 21 (38.9) | 12 (14.8) | 4 (25) | 1 (50) | | 50 (35) |
| Nausea | 5 (13.5) | 80 (11) | 19 (11.7) | 1 (10) | 7 (11.9) | 55 (13.3) | 1 (10) | 3 (14.3) | 0 (0) | 3 (6.3) | 1 (33.3) | 3 (5.6) | 4 (4.9) | 2 (12.5) | 1 (50) | | 21 (14.7) |
| Body temperature increased^+^ | 3 (8.1) | 29 (4) | 2 (1.2) | 0 (0) | 5 (8.5) | 55 (13.3) | 1 (10) | 4 (19) | 1 (33.3) | 4 (8.3) | 0 (0) | 10 (18.5) | 2 (2.5) | 1 (6.3) | 0 (0) | | 1 (0.7) |
| Pyrexia^++^ | 2 (5.4) | 97 (13.4) | 9 (5.5) | 1 (10) | 3 (5.1) | 53 (12.8) | 0 (0) | 2 (9.5) | 0 (0) | 3 (6.3) | 1 (33.3) | 8 (14.8) | 3 (3.7) | 3 (18.8) | 0 (0) | | 14 (9.8) |
| Hyperpyrexia^+++^ | 0 (0.0) | 0 (0) | 0 (0) | 0 (0) | 0 (0) | 0 (0) | 0 (0) | 0 (0) | 0 (0) | 0 (0) | 0 (0) | 0 (0) | 0 (0) | 0 (0) | 0 (0) | | 0 (0) |
